# High-resolution 3D ultrastructural analysis of developing mouse neocortex reveals long slender processes of endothelial cells that enter neural cells

**DOI:** 10.3389/fcell.2024.1344734

**Published:** 2024-03-04

**Authors:** Michaela Wilsch-Bräuninger, Jula Peters, Wieland B. Huttner

**Affiliations:** Max-Planck-Institute of Molecular Cell Biology and Genetics, Dresden, Germany

**Keywords:** cortical development, neurogenesis, radial glial cells, apical progenitors, basal progenitors, endothelial tip cell, sprouting angiogenesis, volume electron microscopy

## Abstract

The development of the neocortex involves an interplay between neural cells and the vasculature. However, little is known about this interplay at the ultrastructural level. To gain a 3D insight into the ultrastructure of the developing neocortex, we have analyzed the embryonic mouse neocortex by serial block-face scanning electron microscopy (SBF-SEM). In this study, we report a first set of findings that focus on the interaction of blood vessels, notably endothelial tip cells (ETCs), and the neural cells in this tissue. A key observation was that the processes of ETCs, located either in the ventricular zone (VZ) or subventricular zone (SVZ)/intermediate zone (IZ), can enter, traverse the cytoplasm, and even exit via deep plasma membrane invaginations of the host cells, including apical progenitors (APs), basal progenitors (BPs), and newborn neurons. More than half of the ETC processes were found to enter the neural cells. Striking examples of this ETC process “invasion” were (i) protrusions of apical progenitors or newborn basal progenitors into the ventricular lumen that contained an ETC process inside and (ii) ETC process-containing protrusions of neurons that penetrated other neurons. Our observations reveal a — so far unknown — complexity of the ETC–neural cell interaction.

## 1 Introduction

The cells in the developing neocortex belong to two principal categories: (i) neural cells and (ii) cells of the vasculature. Regarding the neural cells, two main classes of neural stem and progenitor cells can be distinguished, referred to as apical progenitors (APs) and basal progenitors (BPs), respectively (Taverna et al., 2014). APs reside, and undergo mitosis, in the ventricular zone (VZ), the primary germinal layer and apical-most zone of the developing neocortex. They are characterized by an apical plasma membrane with an apical primary cilium at the ventricular surface and extend basally, often up to the basal lamina. They undergo mitosis at the ventricular surface. In contrast, BPs reside, and undergo mitosis, in the subventricular zone (SVZ), a secondary germinal layer located basal to the VZ. During neocortex development, APs generate mostly BPs (a process called indirect neurogenesis). However, depending on the brain region and species, APs can also generate neurons rather than BPs (a process called direct neurogenesis). The neurons thus generated delaminate from the VZ and migrate, through the intermediate zone (IZ), to the cortical plate (CP) (Cardenas et al., 2018; Cardenas and Borrell, 2020). The newborn BPs also delaminate from the VZ but migrate only to the SVZ, where, depending on the mammalian species, they undergo a varying degree of self-amplification, followed by the generation of neurons and later of macroglial cells (astrocytes and oligodendrocytes; a process called gliogenesis) (Kriegstein and Alvarez-Buylla, 2009; Taverna et al., 2014; Tabata, 2015; Ohtsuka and Kageyama, 2019; Clavreul et al., 2022).

Regarding the cells of the vasculature in the developing neocortex, there are two major classes: (i) endothelial cells and (ii) mural cells (pericytes and smooth muscle cells) (Gerhardt and Betsholtz, 2003; Daneman et al., 2010; Bjornsson et al., 2015; Vanlandewijck et al., 2018; Siekmann, 2023). Endothelial tip cells (ETCs) are a distinct subtype of endothelial cells (Gerhardt et al., 2003; De Smet et al., 2009; Eilken and Adams, 2010). ETCs drive the extension of the blood vessels from pre-existing blood vessels through a process called sprouting angiogenesis, which is the major mode of vascularization in the developing neocortex (Risau, 1997). ETCs extend long filopodia (Marin-Padilla, 1985; Gerhardt et al., 2003; De Smet et al., 2009), through which they explore the environment and migrate in the direction of further blood vessel growth. The blood vessel extension is achieved by proliferation of the endothelial stalk cells, as endothelial tip cells are non-proliferative. This phase of blood vessel sprouting, in combination with branching and anastomosis, is followed by adaptive remodeling and maintenance to refine the blood flow for the later phase of neurogenesis (De Smet et al., 2009; Eilken and Adams, 2010; Potente et al., 2011).

The interaction of the vasculature with the neural cells in the developing neocortex has great functional relevance. Thus, oxygen tension has been shown to be a crucial modulator of cortical development (Wagenfuhr et al., 2015; Lange et al., 2016; Markert and Storch, 2022), with hyperoxygenation driving both proliferation and differentiation of neural progenitor cells. In addition to this effect of hyperoxygenation on progenitors in the direct vicinity of blood vessels, an avascular zone in the VZ has been shown to be important for correct brain development by induction of HIF-1α and VEGF expression (Tomita et al., 2003; Mackenzie and Ruhrberg, 2012; Komabayashi-Suzuki et al., 2019). This, in turn, induces downstream signaling cascades, which are important for the proliferation of both APs and endothelial cells (Santhosh and Huang, 2015; Paredes et al., 2018; Helker et al., 2020). In addition, the growing thickness and cell packing of the cortical wall during development put constraints on the oxygenation of the tissue, which are compensated by increased vascularization of the neocortex with the progression of development. Vascularization of the neocortex starts at the same time as neurogenesis (at E9–10 in the mouse embryo) by ingrowth of initial blood vessels from a pial vessel in the ventral telencephalon (Vasudevan and Bhide, 2008; Bjornsson et al., 2015). Subsequently, these initial blood vessels branch out in a ventral-to-dorsal gradient to form a periventricular vascular plexus (Marin-Padilla, 1985; Vasudevan et al., 2008; Kurz, 2009). From this initial radial vessel outgrowth, tangential connections form, by E14 in the mouse embryo, two major plexi: (i) a meshwork of hexagonally arranged blood vessel branches in the VZ/SVZ and (ii) a second vessel branch at the interface between IZ and CP (Stubbs et al., 2009; Komabayashi-Suzuki et al., 2019).

In further support of the functional relevance of the interaction of the vasculature with neural progenitor cells in the developing neocortex, intermediate progenitors are often associated with, and divide next to, blood vessel branches in the developing SVZ (Javaherian and Kriegstein, 2009; Stubbs et al., 2009; Tan et al., 2016). Moreover, mitotic APs have been shown to be contacted by filopodia emerging from endothelial cells (Komabayashi-Suzuki et al., 2019; Di Marco et al., 2020; Penna et al., 2021). A number of these filopodia contacts have been implicated in the regulation of AP cell cycle progression, with consequences for neurogenesis (Di Marco et al., 2020). In addition, it has been shown that endothelial cells can influence, in co-cultures, progenitor self-renewal and expansion (Shen et al., 2004). Moreover, endothelial cell stabilization and vessel sprouting are influenced by mural cells (summarized in Siekmann (2023)). In addition, endothelial cell cultures or conditioned medium can modulate the number of neurite outgrowth and spines in maturing neurons (Wu et al., 2017). Conversely, neural cells secrete factors like VEGF which influence angiogenesis (Paredes et al., 2018; Di Marco et al., 2020).

In the present study, we have sought to obtain an insight into the interaction of the vasculature and neural cells in the embryonic mouse neocortex at the ultrastructural level and in 3D, as this insight has been lacking from previous studies. To this end, we have chosen a volume electron microscopy (EM) approach using serial block-face scanning EM. Our key finding is that endothelial cell-derived filopodia can enter and traverse the cytoplasm of neural progenitor cells and neurons, and even exit these cells, in the developing mouse neocortex. These novel forms of endothelial cell–neural cell interactions suggest that developing blood vessels likely have an even more complex impact on the developing neocortex than previously assumed. Moreover, as sprouting angiogenesis is a conserved process, it is conceivable that morphological interactions similar to the ones described here may also exist during the development of other tissues.

## 2 Materials and methods

### 2.1 Tissue

All experiments were conducted in accordance with the Animal Welfare Legislation issued by the German federal government. For the generation of the serial block-face SEM samples, C57BL/6JOlaHsd mice were used. Mice were kept under standard pathogen-free conditions in the animal facility of the Max Planck Institute of Molecular Cell Biology and Genetics, Dresden. All mice were killed by cervical dislocation, the uterus was removed, and embryos were dissected under fixation. Embryos at embryonic day (E) 14.5 were decapitated and the skull carefully removed prior to fixation. For embryos at E12.5, the entire head was cut and fixed. Fixation was conducted in 4% PFA and 1% GA in 0.1 M phosphate buffer, pH 7.2 for 1 h at room temperature, followed by at least 24 h of fixation at 4°C.

### 2.2 Serial block-face scanning electron microscopy

Tissues were fixed as described above and embedded in 4% low-melting agarose (Science Services) in PBS (containing 0.5 mM MgCl_2_ and 0.9 mM CaCl_2_), followed by the preparation of transverse 200-µm vibratome sections (VT1200S, Leica). The 200 µm-sections were post-fixed in 2% osmium tetroxide containing 1.5% potassium ferrocyanide for 30 min at room temperature, stained with 1% thiocarbohydrazide (EMS) for 15 min, fixed with 1% osmium tetroxide for 30 min, stained with 1% thiocarbohydrazide for 20 min, and incubated again with 1% osmium tetroxide for 30 min. Samples were subsequently contrasted with 0.5% uranyl acetate in 25% methanol overnight at 4°C and with Walton’s lead acetate for 30 min at 60°C. After ethanol dehydration, the samples were infiltrated and flat-embedded in Epon replacement (Carl Roth). Details on sample preparation have previously been described (Paridaen et al., 2015).

A small portion of the resin-embedded dorsal telencephalon was mounted on a pin, pre-trimmed in a microtome, quality-controlled by ultrathin sections in a standard TEM (Thermo Fisher/FEI, Morgagni at 80 kV with EMSIS, MORADA camera), and placed in a high-vacuum scanning electron microscope (Thermo Fisher Scientific/FEI, Magellan 400) equipped with an internal microtome (AMETEK/Gatan, 3ViewXP). The Gatan DigiScan II V BSE detector was used for image recording. Serial sectioning was performed at 50 nm sectioning thickness. Prior to imaging, the sample was allowed to degas for at least one night in the SEM chamber. Serial backscattered electron images of the block face were recorded as multiple (10–30) image tiles, with an overlap of roughly 10% per section.

Two separate datasets were acquired from two tissue pieces taken from two vibratome sections that had undergone the same embedding of an embryonic E14.5 C57BL/6JOlaHsd brain. These two datasets hence represented two distinct regions of an E14.5 mouse neocortex. In addition, we acquired a third dataset from a piece of the E12.5 mouse neocortex (Paridaen et al., 2013; Taverna et al., 2016). The parameters of the three datasets are listed in Table 1.

**TABLE 1:** Parameters of the three SBF-SEM datasets.

	Number of slices	Volume dimensions x/y/z (µm)	Slice thickness (nm)	Pixel size (xy, nm)	Accelerating voltage	Beam current	Tile size (pixels)
E14.5 Dataset 1	1,897	400/115/95	50	7.3 × 7.3	2.0 kV	100 pA	5,800 × 5,800
E14.5 Dataset 2	1,500	370/80/75	50	7.5 × 7.5	1.9 kV	100 pA	5,500 × 5,500
E12.5	1,000	170/300/50	50	9 × 9	1.9 kV	100 pA	5,200 × 4,000

### 2.3 Image processing, segmentation, and quantification

For each of the datasets, the recorded images of tiled layers were compiled into a montage, and the montaged layers of the serial sections were aligned as a volume in the TrakEM2 plugin in Fiji software (Cardona et al., 2012; Schindelin et al., 2012). Montaging of the single tiles within one layer and alignment over all layers was performed by SIFT registration with similarity or affine alignment with a final rigid constraint. For better alignment and tracking, an image filter was applied to the image visualization (Robust Normalize Local Contrast Filter in TrakEM2).

Cell profiles were manually tracked and indicated by color on every third to fifth section layer. Features of interest were marked during tracking and re-evaluated later. Cell profiles at a reasonable spacing were fully colored for better 3D visualization with the 3D-viewer plugin in Fiji using a script provided by A. Cardona. Three-dimensional models in the 3D viewer were rendered at a resampling factor of 25, and views were recorded with automatic 2° rotations along the apical–basal axis. Volume visualization (Figure 3A) was performed in Dragonfly (Object Research System) software on a non-commercial research license.

Cells were classified as apical progenitors if they contained an apical plasma membrane with an apical primary cilium and a process extended toward the basal side. Cells were classified as basal progenitors if they had a basolateral cilium and a process extending toward the basal side. Cells were classified as newborn basal progenitors if they were still integrated into the apical adherens junction belt and had a basolateral cilium. Cells were classified as neurons if they had multiple processes, particularly one very long process (mostly in the lateral direction). Borders between cortical layers were defined according to the arrangement of nuclei. Cells were classified as endothelial cells if they limited a blood vessel lumen in which erythrocytes (identified by higher electron density and homogenous structure) were observed. Cells were classified as endothelial tip cells if they were in tight association with another endothelial cell, irrespective of whether they had contact with the blood vessel lumen or not, and if they exhibited—as a defining criterion—multiple slender processes with a length of more than 10 µm. Cells were classified as mural cells if they were directly adjacent to endothelial cells, were very flat, and had a more electron-dense cytoplasm than surrounding cells. Given the microvessel nature of the blood vessels studied here, we consider most, if not all, mural cells analyzed in this study to be pericytes (Yamazaki and Mukouyama, 2018).

For quantification, processes of ETCs were grouped according to the position of their cell body on a blood vessel in the VZ or in the SVZ/IZ. We quantified filopodia originating at E12.5 from two ETCs in the VZ and at E14.5 from six ETCs in the VZ and four ETCs in the SVZ/IZ in dataset 2 and from three ETCs in the VZ in dataset 1. The analysis of the counted ETC process endings was either performed as a percentage over the total number of processes in all layers or a percentage over the total number of ETCs/processes only in the VZ or SVZ/IZ, respectively. Numbers, which had been determined from the TrakEM2 segmentation, were entered in Prism software for graphical visualization.

### 2.4 Terminology used in this study

We use the term ETC “*filopodia*” for processes in the intercellular space, whereas we use the term ETC “*processes*” when they enter in cells.

To describe the mode of the interaction of ETC processes with other cells, we use the following terms:

*“enter”* when ETC processes are engulfed by the host cell plasma membrane and cytoplasm;*“traverse”* when ETC processes run within the host cell cytoplasm for a long stretch, but end within the host cell; *“penetrate”* when ETC processes enter, traverse, and then exit the host cell.

We use the term “*chimeric process*” for a protrusion of a host cell fully surrounding an ETC process in a third compartment (either cell or ventricle).

## 3 Results

### 3.1 Endothelial tip cells extend their processes through the ventricular zone of the embryonic mouse neocortex

To obtain a novel insight into the morphology of the developing neocortex, we have performed a high-resolution, 3D electron microscopic study to reveal the specific cellular and subcellular features of the various cell types in the embryonic mouse neocortical wall at the ultrastructural level. To this end, we chose serial block-face scanning electron microscopy (SBF-SEM) as the approach to volume imaging in order to generate a dataset that is large enough to encompass entire cells (Denk and Horstmann, 2004; Titze and Genoud, 2016). The volume acquired from mouse embryonic day (E) 14.5 dorsal telencephalon spanned 115 × 400 × 95 µm (lateral dimension x radial dimension x stack thickness, respectively), with a pixel resolution of 7.3 × 7.3 nm in xy and 50 nm in z (for details, see Methods). This dataset allowed us to track individual cells in their entirety, from apical to basal. Comparison of the outline of the individual tracked cells with available light microscopy data allowed us to assign each to an established cell type (Taverna et al., 2014). Importantly, the dataset provided detailed, 3D ultrastructure information about the tracked cells in the context of their tissue.

**FIGURE 1:**
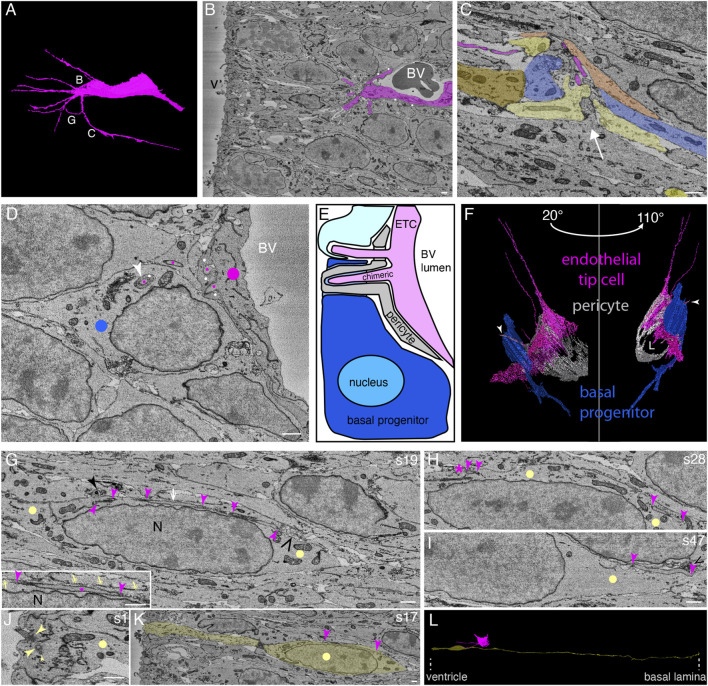
In the embryonic mouse neocortex, endothelial tip cell processes can enter, traverse the cytoplasm, and even exit neural cells in the ventricular zone, with cytoplasmic vesicle clusters often associated with the endothelial tip process. **(A)** Model of an apical ETC from a segmented serial block-face SEM volume through the ventricular zone of a mouse E14.5 neocortex with many long, slender filopodia originating from the cell body. The white letters indicate the positions from which the images in the corresponding subsequent panels are acquired. **(B)** Single plane of the SBF-SEM stack through the apical ventricular zone of the mouse E14.5 neocortex showing the extremity of a blood vessel (BV, with a dark erythrocyte in the lumen) surrounded by an ETC (colored in magenta, model view in (A)) with multiple filopodia and processes. White dots indicate a pericyte adjacent to the ETC. v, ventricle. **(C)** Apical progenitors (each AP is indicated by a different type of yellow color) and a newborn basal progenitor (blue) in the vicinity of an endothelial tip cell filopodium (magenta, originating from the blood vessel (not shown) located at the upper side of the micrograph) in the apical ventricular zone. The neural cells adjust their shape and extend protrusions toward the tip cell filopodia (the white arrow indicates the direction of the protrusions). **(D)** Single plane of the SBF-SEM stack showing an ETC (marked by magenta dots) delimiting a blood vessel (BV) and extending processes that traverse the neighboring pericyte (marked by white dots) and enter the neighboring progenitor cells (blue dot: BP), either on their own or ensheathed by a protrusion of the pericyte (white arrowhead). **(E)** Schematic representation summarizing the position shown in **(D)**. The processes of the ETC (magenta) are traversing the pericyte and entering the neighboring progenitor cells (progenitor: turquoise and newborn BP: blue), either on their own (upper process) or traversing the cytoplasm surrounded by a chimeric, sheath-like process of a pericyte (lower process, pericyte: gray). The cells entered by the ETC process are a pericyte (gray), a newborn basal (blue) progenitor, and a progenitor (not defined whether apical or basal, light turquoise). BV lumen, blood vessel lumen. **(F)** Model from the SBF-SEM segmentation of the position shown in **(D)**. The ETC (magenta) entering a pericyte (white) into a newborn basal progenitor (blue) in views at two different angles (20° and 110° tilts with respect to the plane of **(D)**). The white arrowhead is pointing to the penetrating chimeric process. L, blood vessel lumen. **(G–I)** Selected SBF-SEM planes through an apical progenitor cell (yellow dots) in the apical VZ traversed by an ETC process (indicated by magenta arrowheads). The ETC process is passing through the elongated progenitor nucleus (N). The area indicated by the white arrow in **(G)** is shown enlarged in the inset at the bottom left. There, the plasma membrane of the surrounding progenitor cell is marked with yellow double-headed arrows. The solid black arrowhead points to a vesicle cluster bulging from the AP cytoplasm into a neighboring cell. The open black arrowhead points to a vesicle cluster within the AP cytoplasm. **(H, I)** Different sectioning planes (as indicated by the section number (s) on the top right) from **(G)** show the endothelial process entering (I, s47) and terminating in (H, s28, asterisk) the apical progenitor cell. Vesicle clusters close to the entry point of the endothelial process in the progenitor cells are indicated by black open arrowheads in **(G)** and **(I)**. A vesicle cluster protruding from the apical progenitor and surrounded by a neighboring cell is indicated by a black closed arrowhead in **(G)** (right). **(J)** First sectioning plane (s1) showing the apical-most end of the cell shown in **(G)**, with an apical primary cilium emerging from a basal body and a duplicated centriole (large arrowheads, basal body, and mother centriole; small arrowhead, duplicated daughter centriole). **(K)** Sectioning plane through the apical-most VZ, showing the apical process and the nuclear region of the cell shown in **(G)** (indicated in yellow). The ETC process shown in **(G)** is visible between the two arrowheads. **(L)** Model view of the AP (yellow) entered by the ETC (magenta) process shown in panels **(G–K)** from the segmented SBF-SEM stack. The position of the ventricular surface and the basal lamina is indicated by dotted lines at the bottom. The scale bar for all panels in Figure 1 is 1 µm. Dots, arrows, and arrowheads indicate structures of the following cell types: endothelial cell (magenta), AP (yellow), BP (blue), and pericyte (white).

In the course of this analysis, we identified neural cell types—apical radial glia, BPs, and neurons—as well as cells of the growing vasculature of the tissue. Blood vessels, sometimes containing erythrocytes, were easily detectable (see Figure 1B and Supplementary Video S1 for more sectioning planes of the SBF-SEM stack). The blood vessels were surrounded by endothelial cells, which, in some cases, were partially covered by pericytes (Figure 1B and Supplementary Video S1). Some endothelial cells extended slender, very long filopodia into the surrounding tissue (Figures 1A, B and Supplementary Video S1, Supplementary Presentation S1, Models to Figure 1). Such long processes are typical of endothelial tip cells, a cell type which is known to drive sprouting angiogenesis (Gerhardt et al., 2003; De Smet et al., 2009). These endothelial cells not only sprouted into the surrounding tissue but also delimited the lumen of the blood vessel at its distal end (Figures 1B, D and corresponding Supplementary Videos S1, S2). Despite the latter finding, we shall regard these endothelial cells that extend slender, very long filopodia as endothelial tip cells (ETCs; see also Discussion).

In light of the importance of the interplay between neural cells and vascular cells for the development of the neocortex (Walchli et al., 2015; Paredes et al., 2018; Takashima et al., 2019; Di Marco et al., 2020), we sought to obtain further insight, at the single-cell level, into key morphological features that characterize the interaction between these cells. Herein, a particular focus was on the filopodia of ETCs that extend into the surrounding neural tissue.

### 3.2 Endothelial tip cell processes can enter, traverse the cytoplasm, and even exit apical progenitors and basal progenitors

Filopodia of ETCs have been reported to contact the plasma membrane of mitotic APs in the developing brain (Komabayashi-Suzuki et al., 2019; Di Marco et al., 2020; Penna et al., 2021). The subcellular resolution provided by our volume EM approach led to an unexpected, key observation of the present study, namely, that the filopodia of ETCs often entered adjacent cells via deep plasma membrane invaginations of the latter, rather than being only found in the intercellular space. As observed in the VZ, the filopodia either directly entered a neighboring progenitor cell or first entered an adjacent pericyte and then—in either of the following two ways—entered the neighboring progenitor cell. One way was that the ETC filopodium (from now on also referred to as the ETC process) penetrated the pericyte and then entered the progenitor cells, as shown for a progenitor cell in Figure 1D (see the upper, light turquoise cell in Figure 1E for a schematic summary of the cellular interactions, and Supplementary Video S2 and Supplementary Presentation S1, Models to Figure 1). The other way was that the ETC process remained inside a pericyte protrusion, which then entered the progenitor cells, as shown for a BP in Figure 1D (see Figure 1E (dark blue cell) for a schematic summary and Figure 1F for two views at different tilt; the BP was identified as such by tracking the entire cell; see also Figure 1F and the corresponding Supplementary Video S2, Supplementary Presentation S1, Models to Figure 1). We shall refer to such an ETC process-containing protrusion of another cell type (here a pericyte) as a “chimeric” process.

The ETC filopodia were found to enter, traverse, and even exit not only neural cells that were adjacent to vascular cells (i.e., endothelial cells and pericytes) but also neural cells without contact with vascular cell bodies. In the latter case, the ETC filopodia had either first traversed vascular cell-contacting neural cells before entering the neural cells lacking contact with vascular cell bodies, or the ETC filopodia had passed through the intercellular space before entering the neural cells lacking contact with vascular cell bodies. An example of such a progenitor in the VZ that lacked contact to vascular cell bodies is shown in Figures 1G–I (see Supplementary Video S3 and Supplementary Presentation S1, Models to Figure 1 for 3D views). This progenitor was identified as an AP, as it fulfilled the relevant criteria such as an apical cilium and basal lamina contact upon tracking the entire cell (Figures 1J–L). An ETC process with a diameter of 200–300 nm entered the cytoplasm of the AP for a long stretch (>25 µm) parallel to the elongated progenitor nucleus before ending within the progenitor cytoplasm close to the plasma membrane (Figures 1G–I, Supplementary Video S3, and Supplementary Presentation S1, Models to Figure 1).

In addition to our central observation that ETC processes can enter, traverse, and even exit other cells, we noticed that neural cells in the VZ that were located in the vicinity of ETC processes seemed to adjust their morphology in response to the presence of the ETC processes. Specifically, one overt morphological change was that the neural cells extended cytoplasmic protrusions toward these processes (Figure 1C, protrusions in the direction of the white arrow, and Supplementary Video S4 and Supplementary Presentation S1, Models to Figure 1).

Two different types of blood vessel patterns have been described for the developing mammalian cortex: first, sparse blood vessels arranged parallel to the apical–basal axis of the cortical wall, as seen in the cortical plate; second, a higher level of vascularization starting in the basal region of the VZ and extending into the intermediate zone (Vasudevan and Bhide, 2008; Komabayashi-Suzuki et al., 2019). The latter pattern of vascularization can be expected to be associated with sprouting angiogenesis and thus with a high occurrence of ETCs, as this is known to be particularly prominent in the apical region of the VZ and the intermediate zone at the developmental stage studied here. As we have shown that the ETC processes can enter progenitor cells (both APs and newborn BPs) in the VZ, the likely increased abundance of ETCs in the intermediate zone prompted us to investigate whether ETC processes can also enter neural cells in this more basal zone of the developing neocortex.

### 3.3 Endothelial tip cell processes can enter, traverse the cytoplasm, and even exit basal progenitors and newborn neurons in the subventricular and intermediate zones of the embryonic mouse neocortex

**FIGURE 2:**
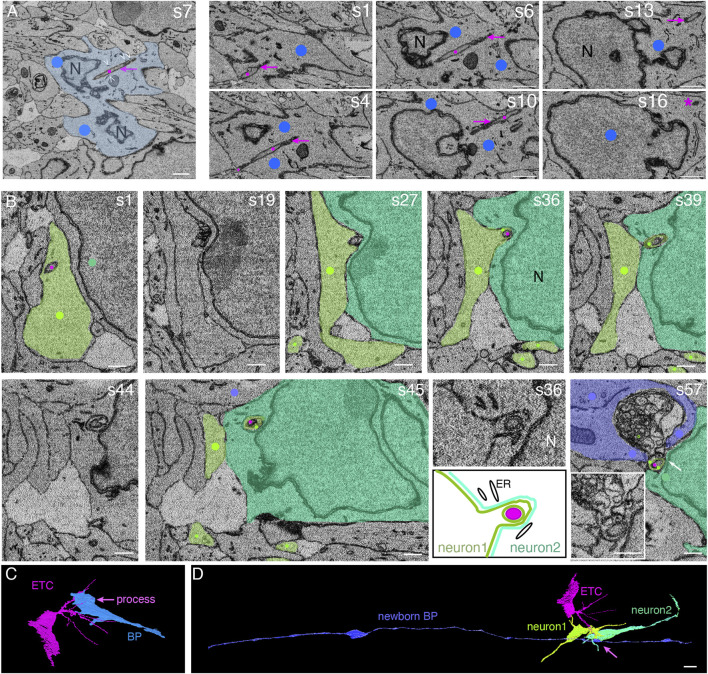
Endothelial tip cell processes can enter, traverse the cytoplasm, and even exit basal progenitors and newborn neurons in the intermediate zone of the embryonic mouse neocortex. **(A)** Selected sectioning planes from a SBF-SEM stack through a basal progenitor (indicated by blue overlay and dots) in the intermediate zone of an embryonic mouse E14.5 neocortex. An ETC process (indicated by magenta dots and arrows) traverses the cytoplasm of the basal progenitor. The ETC process ends in the cytoplasm of the BP close to the plasma membrane opposite to the entry point (asterisk on s16). White open arrowheads in the first panel indicate the endoplasmic reticulum adjacent to the ETC process. Section numbers are indicated at the top right corner. Scale bars: 1 µm. **(B)** Selected SBF-SEM sectioning planes through the intermediate zone of the E14.5 mouse telencephalon. A process from a basal endothelial tip cell (magenta dots) is entering a first neuron (light green) and traversing its cytoplasm (s1–s27). The cytoplasm of the first neuron bulges out and forms a chimeric, sheath-like protrusion surrounding the ETC process, penetrating together the neighboring second neuron (darker green, s36–s45). The end of the ETC process is found in a large cytoplasmic vesicle cluster that is engulfed by a newborn basal progenitor cell (blue, s57). This transition between neuron 2 and the vesicle cluster (marked by the white arrow) is shown at a higher magnification in the inset in panel s57. The sheath-like protrusion of the first neuron into the second neuron shown in s36 is displayed at higher magnification and with a schematic representation below panel s36. ER, endoplasmic reticulum; N, nucleus. Scale bars: 1 µm. **(C)** Model view of the position shown in panel **(A)** from the segmented SBF-SEM stack: the ETC (magenta) traverses with one of its processes the basal progenitor (BP, blue). **(D)** Model view of the position shown in panel **(B)** from the segmented SBF-SEM stack: the ETC (magenta, same cell as in **(C)**) process traverses the cytoplasm of two neurons (greens) and ends engulfed by a newborn basal progenitor (blue) with contact to the ventricle (left) and the basal lamina (right).

We identified ETCs at specific sites of the blood vessels in the intermediate zone, which presumably represented future branching points. These ETCs extended long slender processes with a diameter of ∼200 nm into the surrounding tissue. Some of these long, slender processes entered neural cells in the intermediate zone and extended through most of their cytoplasm, engulfed by deep plasma membrane invagination of the neural cell (Figure 2), similar to ETC processes that entered neural cells in the VZ. The neural cells with entered ETC processes were either BPs (Figures 2A, C and Supplementary Presentation S1, Models to Figure 2) or neurons (Figures 2B, D and Supplementary Presentation S1, Models to Figure 2), as judged from their overall shape, with both lacking contact to the ventricle or the basal lamina.

In the example of the BP shown in Figures 2A, C, the entering ETC process extended from one side of the progenitor cell to the other side. There, the ETC process ended close to the plasma membrane of the progenitor cell (Figure 2A, s16, asterisk, and Supplementary Video S5). On its course through the cell, the ETC process traversed the cytoplasm in close vicinity to the nuclear envelope. Endoplasmic reticulum tubules were often observed in the immediate vicinity of the ETC process (Figure 2A, white open arrowheads and Supplementary Video S5).

ETC processes were also observed to enter and traverse the cytoplasm of neurons in the intermediate zone. A striking example where two neurons are involved is shown in Figure 2B and Supplementary Video S6. Here, an ETC process entered a newborn pyramidal neuron, referred to as neuron #1. The ETC process traversed the cytoplasm of this neuron for approximately 5 μm, contacting endoplasmic reticulum tubules multiple times on its way (Figure 2B; s1 and s19). Opposite to the site of the neuronal cell body where the ETC process had entered, the neuronal cytoplasm and plasma membrane bulged out (Figure 2B; s27 and s36) and, ensheathing the ETC process, formed a chimeric process that entered another neuron, referred to as neuron #2; traversed its cytoplasm for ≈3 μm; and eventually exited neuron #2 (Figure 2B; s39–s57, and Supplementary Video S6 for the full SBF-SEM stack).

### 3.4 Cytoplasmic vesicle clusters are often associated with intracellular endothelial tip cell processes

In addition to the key observation that ETC processes can enter, traverse, and even exit neural cells, we noticed that the intracellular ETC processes were often associated with clusters of vesicles in the cytoplasm of the neural “host” cell. We shall describe the detailed features of this association in two examples: (i) the neuron #1, shown in Figure 2B and (ii) the AP, shown in Figures 1G–I.

With regard to the chimeric process of neuron #1 that contained an ETC process inside and penetrated neuron #2, as this chimeric process emerged from neuron #2, it was associated with a vesicle cluster that was engulfed by yet another cell, a newborn BP (for evidence, see below) (Figure 2B, s57). This vesicle cluster consisted of a dense packing of vesicles with a diameter of 100–300 nm. The cell engulfing the vesicle cluster contained a basolateral cilium in the apical-most region of the lateral plasma membrane, an apical plasma membrane constituting a ventricular surface, and extended a radial process toward the basal lamina (data not shown) (Figure 2D and Supplementary Presentation S1, Models to Figure 2). We therefore classified this vesicle cluster-engulfing cell as a newborn BP.

In the case of the AP shown in Figures 1G–L, clusters of vesicles of the host cell were often observed along the ETC process as it traversed the host cell cytoplasm (Figures 1G, I, black open arrowheads, and Supplementary Video S3). These vesicles were up to 200 nm in diameter. We also noticed such a vesicle cluster as the cytoplasm and plasma membrane of this AP bulged out into the neighboring cell (Figure 1G, black solid arrowhead, and Supplementary Video S3).

### 3.5 Endothelial tip cell processes that have entered apical progenitors in the embryonic mouse neocortex extend as long “chimeric” processes into the ventricle lumen, with cytoplasmic vesicle clusters at the end of the process

**FIGURE 3:**
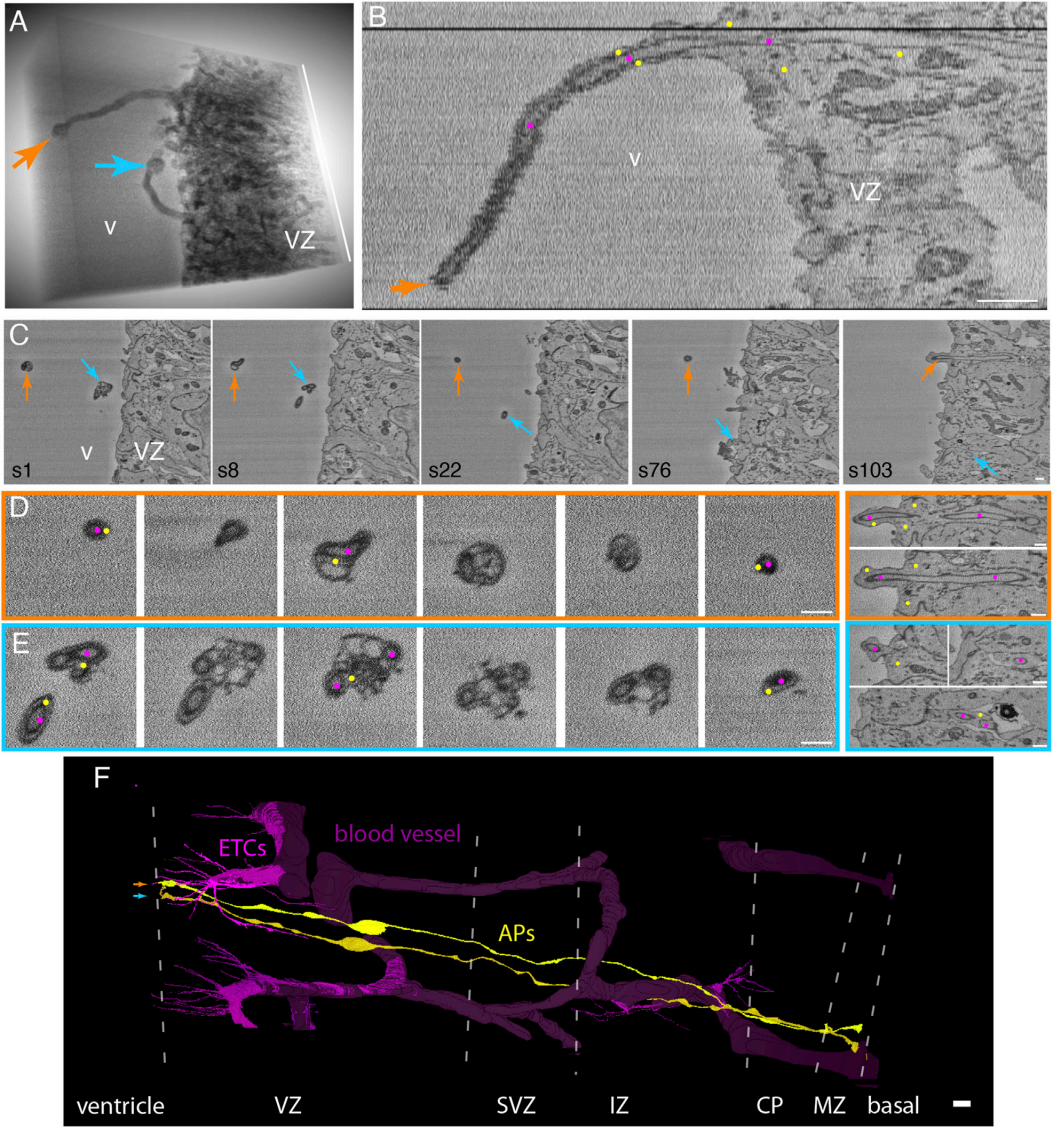
Endothelial tip cell processes that have entered apical progenitors in the embryonic mouse neocortex extend as long “chimeric” processes into the ventricle lumen, with cytoplasmic vesicle clusters at the process. **(A)** 3D representation of an SBF-SEM volume through the apical-most VZ of a mouse E14.5 neocortex (to the right) with two processes (orange and blue arrows) protruding into the ventricle (v). The processes end in bubble-like structures. The height of the volume is 12.6 µm (white bar on the right). **(B)** Single xz-plane representation of the SBF-SEM stack containing the orange ventricular protrusion shown in panel **(A)**. The longitudinal view through the protrusion reveals that it consists of an ETC process (magenta dots) surrounded by the cytoplasm sheath of an apical progenitor (AP, marked by yellow dots). **(C)** Selected sectioning planes (section numbers (s) indicated at the bottom left) of the volume shown in **(A)** with cross-sections through the two ventricular protrusions (orange and blue arrows). **(D, E)** High-magnification selected sectioning planes (in ∼250-nm steps) through the orange and blue chimeric processes in the ventricular lumen (left boxed panels) and of the apical-most stretch of the VZ (right boxed panels). The color of the frames indicates the respective labeled protrusion in **(A)** and **(C)**. These cross-sections show the clustered vesicles in the AP cytoplasm (yellow dots) surrounding the ETC processes (magenta dots) at the bubble-like end of the protrusion. The ETC processes can be followed through the apical end-feet of the respective APs (right panels). v, ventricle; VZ, ventricular zone. Scale bars in **(B–E)** are 1 µm. **(F)** Model view of the endothelial tip cells (magenta) and blood vessels (dark violet) contained in the SBF-SEM volume through the mouse E14.5 neocortex. The apical progenitor cells labeled in orange and blue (in A-E) in which the ETC (magenta) processes traversing are shown in yellow. They span from the ventricle (left) to the basal lamina (right). The positions of the borders between the cortical layers are indicated by dotted white lines. VZ, ventricular zone; SVZ, subventricular zone; IZ, intermediate zone; CP, cortical plate; MZ, mantle zone. The scale bar in **(F)** is 10 µm.

We observed a remarkable feature of ETC processes inside neural progenitors in the VZ, for which two examples are shown in Figure 3. Specifically, ETC processes, after having traversed the cytoplasm of apically attached progenitor cells, extended as a chimeric process into the ventricular lumen, with the ETC process (magenta dots) being ensheathed by a long, thin protrusion of the progenitor cell (yellow dots, Figure 3 and Supplementary Video S7, and another example from ultrathin transmission EM sections in Supplementary Presentation S1, Image to Figure 3). In one example, the chimeric process extended for up to 9 µm into the ventricular lumen (Figures 3A, B; magenta dots in B label the ETC and yellow dots label the AP sheath). Of note, at the end of the progenitor protrusion, an accumulation of vesicles (100–300 nm in diameter), apparently associated with the tip of the ETC process, formed a bubble-like structure (Figures 3D, E and Supplementary Video S7). The diameter of the ETC process inside the progenitor protrusion was similar to that in the progenitor cell body (≈200 nm). In addition to the ETC process, very few, if any, cytoplasmic structures of the progenitor cell were detectable within the chimeric processes. The progenitor cells shown in the two examples in Figure 3 extended from the ventricular lumen to the basal lamina (Figure 3F and Supplementary Presentation S1, Model to Figure 3) and contained an apical primary cilium (Supplementary Video S7), indicative of APs.

### 3.6 Endothelial tip cell processes can enter mitotic apical progenitors in the embryonic mouse neocortex

**FIGURE 4:**
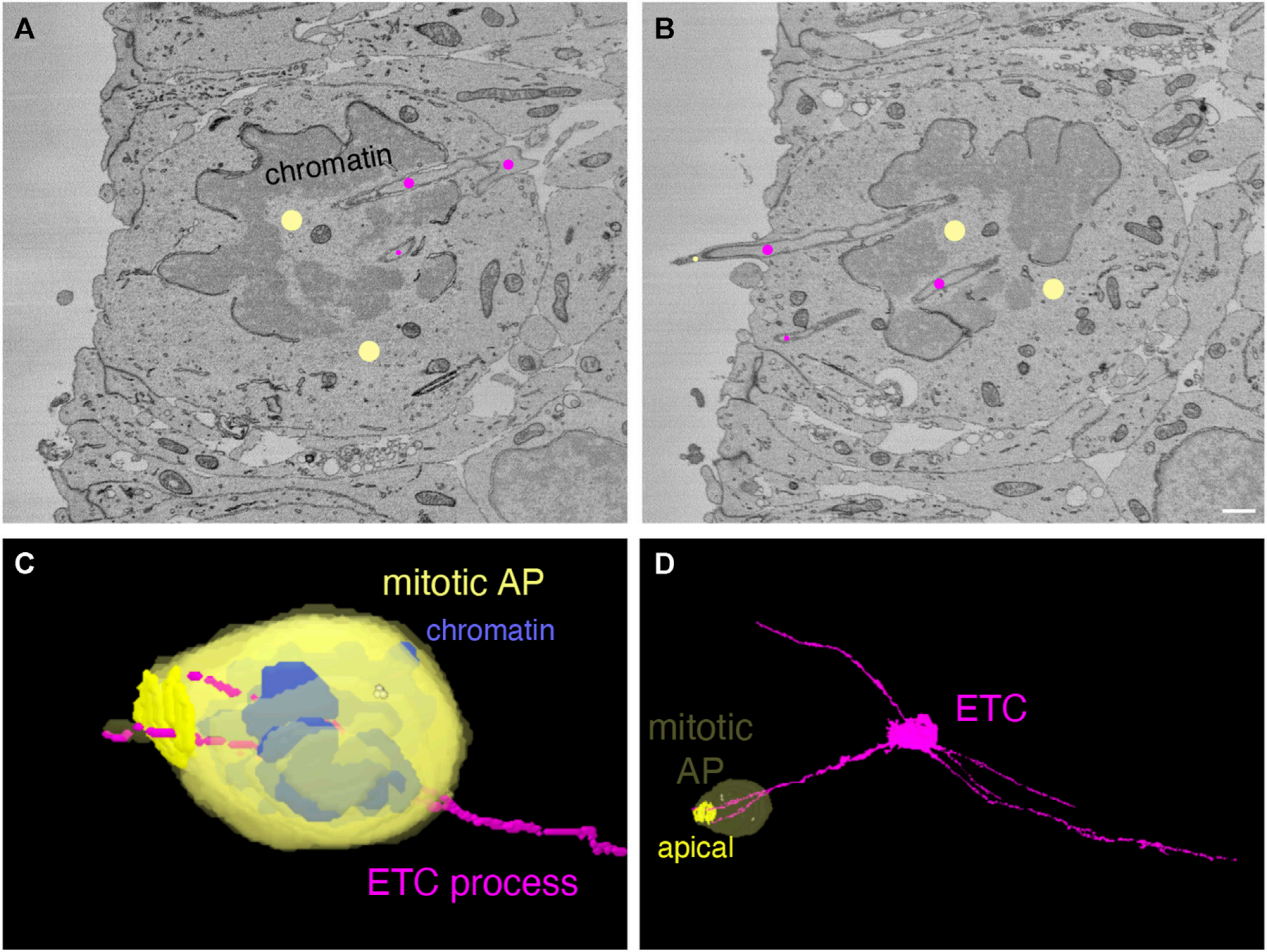
Endothelial tip cell processes can enter mitotic apical progenitors in the embryonic mouse neocortex. **(A, B)** Two sectioning planes through an anaphase cell (marked by yellow dots) at the ventricle surface of a mouse E14.5 neocortex serial block-face SEM volume. The anaphase cell is traversed by ETC processes (marked by magenta dots). An ETC process is splitting at the basal end of the anaphase cell (in A) into two branches that pass through the de-condensing chromatin. One ETC branch ends within the cytoplasm of the mitotic cell, whereas the other branch extends into the ventricle surrounded by a protrusion from the anaphase cell. The scale bar in **(B)** (for A + B) is 1 µm. **(C, D)** Model view from the segmented SBF-SEM stack of the anaphase cell (yellow) shown in (A) and (B) with blue chromatin and the magenta ETC processes. **(C)** Higher magnification. **(D)** Overview. The apical plasma membrane of the anaphase cell is highlighted in (D). The centrioles are indicated as yellow dots within the anaphase cytoplasm.

All the data presented so far regarding our finding that ETC processes can enter, traverse the cytoplasm, and even exit neural cells pertained to cells in interphase. Two reports have provided compelling evidence that blood vessel endothelial cells in developing rodent brain, and specifically the neocortex, extend fine processes that directly contact mitotic neural progenitor cells (Komabayashi-Suzuki et al., 2019; Di Marco et al., 2020; Penna et al., 2021). In light of our data of APs and BPs in interphase, we wondered whether ETC processes may also enter mitotic neural progenitor cells. To explore this possibility, we obtained an additional dataset of E14.5 mouse dorsal telencephalon for SBF-SEM to increase the probability of finding mitotic neural progenitor cells with intracellular ETC processes (80 × 370 × 75 μm, lateral dimension x radial dimension x stack thickness, respectively, pixel resolution of 7.5 × 7.5 nm in xy and 50 nm in z (for details, see Methods). Indeed, in this additional 3D dataset, we observed that a cell undergoing mitosis at the ventricular surface, i.e., a mitotic AP, was entered by two processes of an ETC localized in the VZ (see Figure 4, panels A and B for two sectioning planes at the ventricular surface (see Supplementary Video S8 for the full SBF-SEM stack through the cell) and panels C and D for overviews of the 2 cells concerned (Supplementary Presentation S1, Models to Figure 4)). Hence, ETC processes can enter mitotic APs.

### 3.7 At least half of all endothelial tip cell processes in the ventricular zone and intermediate zone of the embryonic mouse neocortex enter neural cells

**FIGURE 5:**
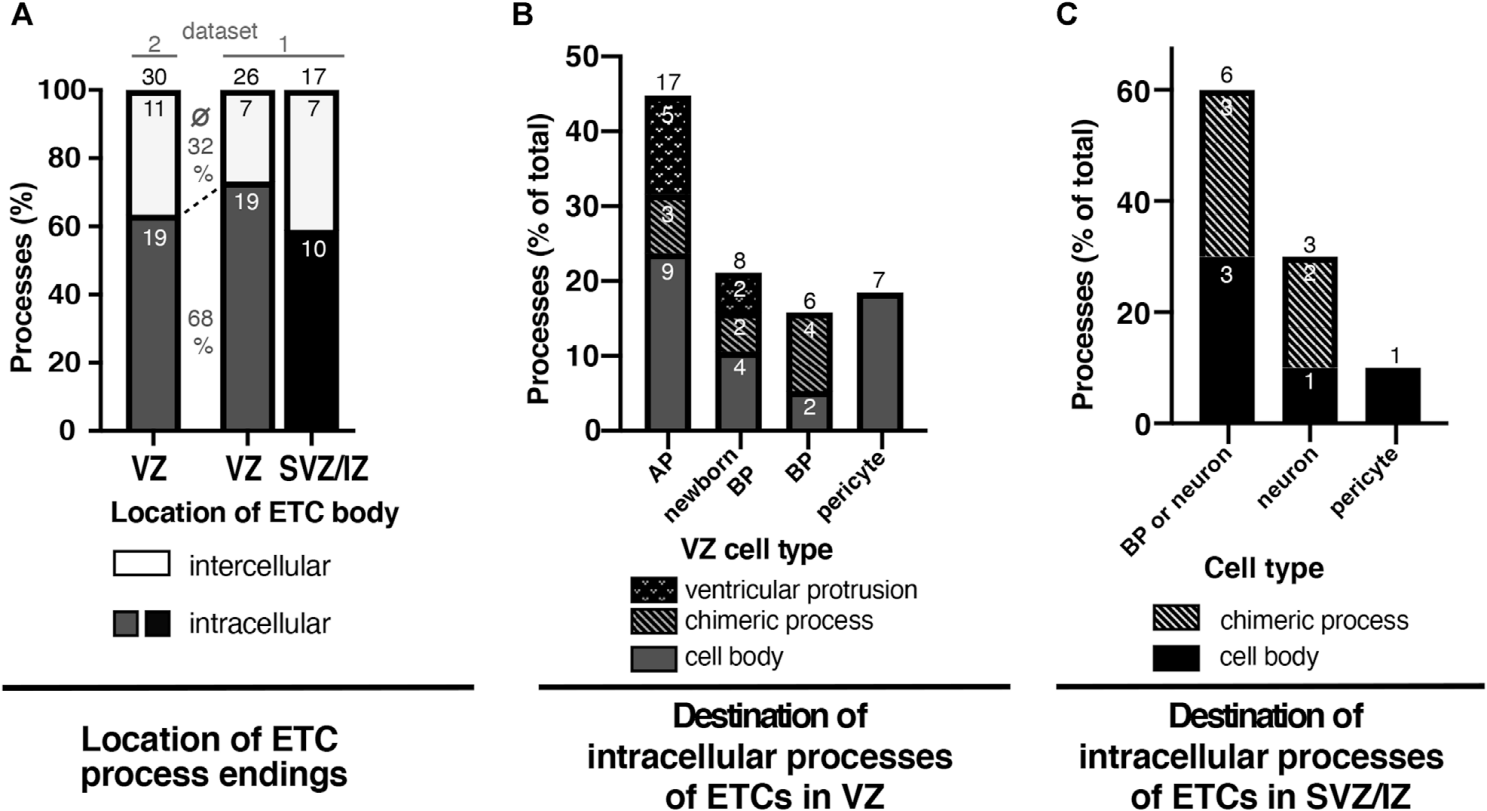
At least half of all ETC processes in the VZ and SVZ/intermediate zones of the E14.5 mouse neocortex enter neural cells. **(A)** Graph depicting the frequency (in %) of ETC filopodia/processes ending in the intercellular space (white) or surrounded by cells (“intracellular”) in the ventricular zone (VZ, dark gray) or the SVZ/intermediate zone (SVZ/IZ, black) of an SBF-SEM stack through the E14.5 embryonic mouse neocortex. The numbers within the bars indicate the actual number of endings analyzed. The black numbers above the bars indicate the total number of endings analyzed for ETCs with cell bodies in the VZ (first and second bars) or in the SVZ/IZ (third bar). The cells analyzed for the first bar are present within dataset 2, as indicated by the line above the bar. Accordingly, the cells analyzed for the second bar (VZ) and third bar (SVZ/IZ) are present in dataset 1. The percentages given between the first and second bars are the average of the percentage of the inter- (top number) and intracellular (bottom) ETC endings in the VZ. **(B)** Graph depicting the frequency (in %) of the intracellular ETC process endings originating from cell bodies in the ventricular zone (dark gray bars in (A)) classified according to the entered cell type: APs, newborn basal progenitors, BPs, or pericytes. The percentage of endings extending with AP protrusions into the ventricular lumen is shown as a dotted sub-area. The percentage of ETC endings traversing as chimeric processes before entering the progenitor cell is indicated by a diagonal pattern. The small white numbers indicate the actual number of endings analyzed. **(C)** Graph depicting the frequency (in %) of the intracellular ETC process endings originating from cell bodies in the subventricular and intermediate zones (black group from (A)) classified according to the entered cell type: BPs, neurons, or pericytes. Cells of the group “basal progenitor or neuron” could not be classified unambiguously to either cell type and were, therefore, kept as a separate group. The small white numbers indicate the actual number of endings analyzed. The numbers above the bars give the total number of cells analyzed for the respective zone or cell type.

We quantitated the frequency of the various locations of the ETC processes. First, we investigated the percentage of ETC processes found in the intercellular space compared to intracellularly within the cytoplasm of cells. More than half of all ETC processes were observed to end intracellularly (Figure 5A and Supplementary Data Sheet S1). Specifically, of the 56 processes analyzed that emerged from ETC bodies located in the VZ, 38 had entered a cell (corresponding to 68%), whereas 18 ended in the intercellular space (32%, Figure 5A). Similarly, of the 17 processes analyzed that emerged from ETC bodies located in the SVZ/IZ, 10 had entered a cell (corresponding to 59%), whereas 7 ended in the intercellular space (41%, Figure 5A) Thus, when quantitating all ETC process endings together, 34% were found in the intercellular space (25 of a total of 73), whereas 66% ended inside a cell cytoplasm (45 of a total of 73).

Second, we quantitated in what cell type and cellular structure the intracellular ETC processes ended in (Figures 5B, C). Thus, we quantitated the intracellular ETC processes originating from ETC bodies residing in the VZ (Figure 5B). The majority (45%) of the 38 ETC process endings analyzed were detected in APs approximately a quarter (21%) in newborn BPs and a slightly smaller number in BPs that had fully delaminated but were still residing in the VZ (16%). A significant proportion of the ETC process endings within APs (5 out of 17 processes) and newborn BPs (2 out of 8 processes, Figure 5B, dotted region of the bar) emerged as a chimeric process into the ventricular lumen. Another significant proportion of the ETC processes penetrated other cells before reaching their final target cell in the VZ (three processes on the way to APs as targets, two on the way to newborn BPs, and four on the way to delaminated BPs; Figure 5B, bar segments with a diagonal pattern). Some ETC processes (three out of nine chimeric processes) even penetrated multiple cells before entering the target cell. These penetrated cells were either progenitors or pericytes (five out of nine ensheathed chimeric processes) on their way to the target cell. A significant number of the ETC endings (18%) were detected within pericytes (7 out of 38 processes in the VZ).

We also quantitated the intracellular ETC processes originating from ETC bodies residing in the SVZ/IZ (Figure 5C). Of the 10 intracellular ETC processes analyzed, the overwhelming majority (7) ended in cells that were either BPs or newborn neurons (Figure 5C). As these cells were not tracked completely, we could not unambiguously identify them as either a BP or a neuron. In addition, all of these cells contained non-duplicated centrioles, which was consistent with them being either BPs in G1 or neurons. Two of the intracellular ETC processes originating from ETC bodies residing in the SVZ/IZ ended in clearly identifiable neurons with long neurites (Figure 5C). One ETC process remained in a perivascular pericyte. Like in the VZ, multiple ETC processes in the SVZ/IZ penetrated cells and formed chimeric processes before entering their target cell (Figure 5C, bar segment with diagonal pattern).

### 3.8 Endothelial tip cell processes can enter and traverse the cytoplasm of apical progenitors and basal progenitors at embryonic day 12.5

**FIGURE 6:**
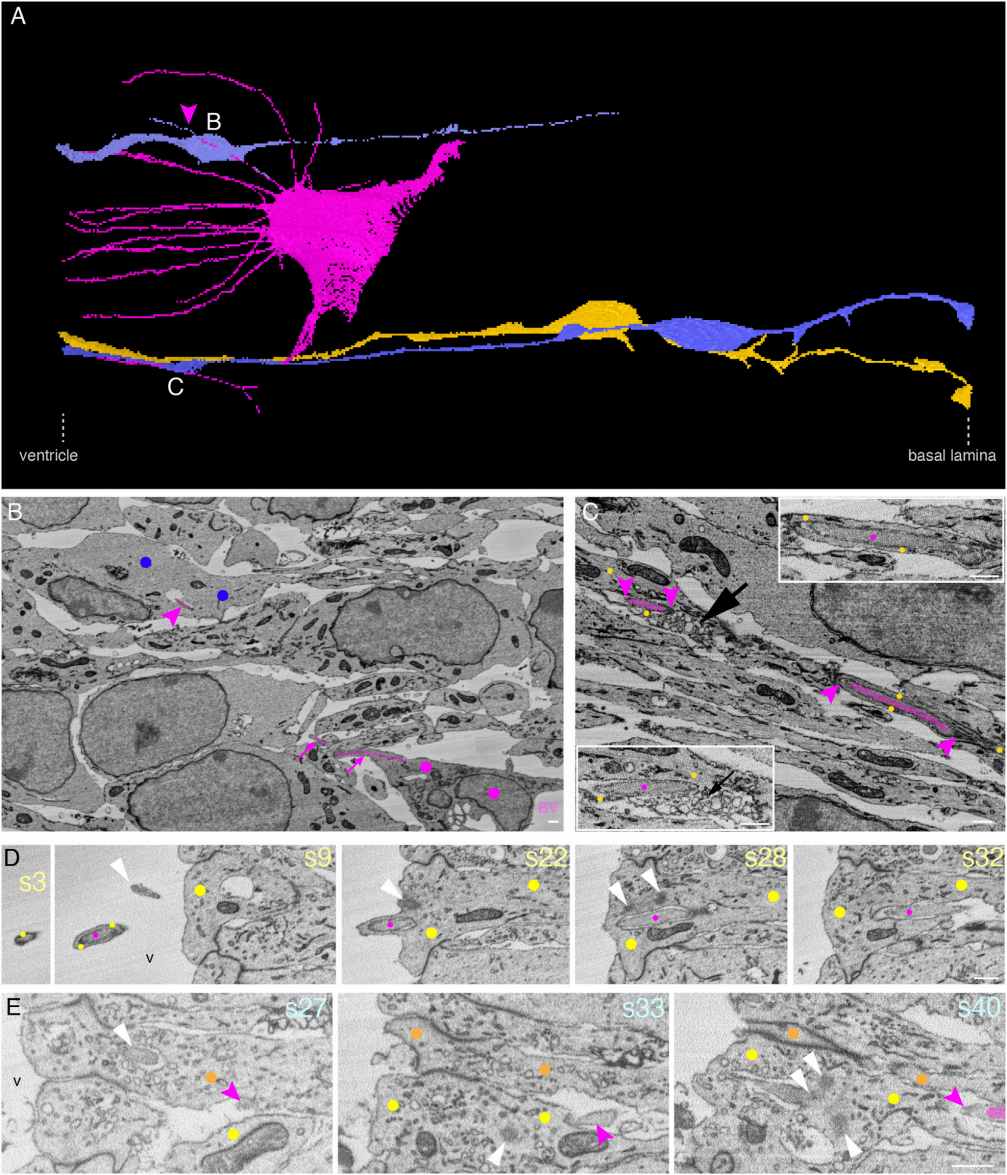
In the E12.5 mouse neocortex, ETC processes can touch the neural cell plasma membrane or enter and traverse the cytoplasm of neural cells in the VZ, with cytoplasmic vesicle clusters associated with the intracellular ETC process. **(A)** Model of an apical ETC and the portion of another ETC from a segmented SBF-SEM volume through the VZ of a mouse E12.5 neocortex, with many long, slender filopodia originating from the cell body. Three progenitor cells (yellow: AP; blue: BPs) which are entered by the ETC processes are shown. The magenta arrowhead points to a chimeric process composed of the ETC process ensheathed by the newborn BP protrusion. The white letters indicate the positions from which the images in the corresponding subsequent panels are acquired. **(B)** Single plane of the SBF-SEM stack through the VZ of the mouse E12.5 neocortex showing the extremity of a BV surrounded by an ETC (colored in magenta, model view in (A)) with a process in this sectioning plane. Part of the process entering the newborn basal progenitor (marked with blue dots) is visible (upper magenta arrowhead). **(C)** Single plane of the SBF-SEM stack through the VZ of the mouse E12.5 neocortex apical to a blood vessel lumen (position as indicated in model view in (A)) with an ETC process (colored in magenta and between magenta arrowheads) traversing the cytoplasm of an AP (indicated by yellow dots). The part of the ETC process on the right is shown at higher magnification in the inset on the top right, and the part on the left in the inset is at the bottom left. A cluster of loosely packed vesicles in the AP cytoplasm next to the ETC process is indicated by the black arrow. **(D)** Selected sectioning planes (section numbers (s) indicated in light yellow at top right) through the apical-most VZ of the mouse E12.5 neocortex with an ETC process (magenta labels) extending into the ventricular lumen (v) surrounded by an AP protrusion (yellow dots). The centrioles (s28), the basal body (s22), and the tip of the apical primary cilium (s9) are indicated by white arrowheads. **(E)** Selected sectioning planes (section numbers (s) indicated in orange at top right) through the apical-most E12.5 VZ (ventricle (v) to the left) with an ETC process (magenta labels) in the intercellular space, terminating on an AP (s27, orange dots) with a primary cilium (white arrowhead in s27) in a deep plasma membrane invagination. The ETC process is touching another AP (s33, yellow dots). Both APs have duplicated centrioles (white arrowheads in s33 and s40). At the site where the ETC process is touching the progenitors, grooves in their plasma membrane are visible. The scale bars for B–E are 1 µm.

Endothelial tip cell filopodia have been reported to contact neural progenitors when blood vessels invade the developing neocortex soon after the onset of neurogenesis (Komabayashi-Suzuki et al., 2019; Di Marco et al., 2020; Penna et al., 2021). Our observation of endothelial tip cell processes not just contacting but actually entering neural progenitors in the mouse neocortex at embryonic day 14.5 prompted us to validate this finding at an earlier developmental stage. We therefore searched a mouse developing telencephalon at embryonic day 12.5 by SBF-SEM for the presence of contacts between ETCs and neural progenitors, in order to then follow the destination of these contacts. Indeed, we detected ETCs extending long, slender processes into the apical VZ (Figure 6A and Supplementary Presentation S1, Model to Figure 6). We detected both (i) processes entering cells (Figure 6B and Supplementary Video S9) and (ii) chimeric processes (Figure 6C, Supplementary Presentation S1, Model to Figure 3 and Supplementary Video S10) consisting of ETC processes ensheathed by the AP cytoplasm. As at E14.5, in the E12.5 VZ, some chimeric processes extended into the ventricular lumen (Figure 6D and Supplementary Video S11).

Quantitative analyses revealed that, when we followed the ETC processes to their end, a significant number of these processes (40%) fully entered cells (10 out of a total of 25 processes analyzed), with the great majority targeting APs (9 out of 10 entering processes). A third of these APs were cells in mitosis. Only one ETC process was observed entering a pericyte, consistent with the low pericyte coverage of the sprouting blood vessels at this developmental stage. One-fifth of all processes analyzed remained entirely in the intercellular space (5 out of a total of 25), whereas twice as many ETC processes ended in the grooves of a progenitor plasma membrane, rather than being fully engulfed by the cell (10 out of a total of 25) (Figure 6E and Supplementary Video S12). Similar to the ETC processes fully entering cells, the majority of the latter processes ended in the grooves of APs (7 out of 10), and only a third ended in the grooves of newborn BPs (3 out of 10). A summary of the number and location of the E12.5 ETC processes is given in Supplementary Data Sheet S1.

As mentioned in section 4.4 above, cytoplasmic vesicle clusters are often associated with intracellular ETC processes at E14.5. We observed similar vesicle clusters at E12.5 in the vicinity of intracellular ETC processes (Figure 6C and Supplementary Video S10), although they were less frequent than at E14.5.

## 4 Discussion

The central findings of our SBF-SEM study in the embryonic mouse neocortex are that filopodia, i.e., long, slender processes, which emerge from endothelial (tip) cells can enter pericytes and neural cells, traverse their cytoplasm, and even exit these cells. We find it likely that these filopodia-extending endothelial cells are, in fact, endothelial tip cells, as the extension of long, slender processes is a characteristic feature of the latter cell type. However, as there is controversy in the literature about whether endothelial tip cells can delimit a vascular lumen (Gerhardt et al., 2003; De Smet et al., 2009), which clearly was the case for the filopodia-extending endothelial cells studied here, we cannot exclude that the latter cells were endothelial cells (rather than endothelial tip cells). These endothelial cells have been reported to also extend long, slender processes that make contact with neural progenitor cells (Penna et al., 2021). However, this does not diminish the novelty of our findings that endothelial cell processes can enter neural cells via deep invaginations of the neural cell plasma membrane.

Although most of the above mentioned central findings were observed upon SBF-SEM analysis of a single E14.5 mouse neocortex, albeit in two different areas of this tissue; these findings are not confined to this one embryo. TEM analysis showed the existence of a chimeric process extending into the ventricular lumen of another E14.5 mouse neocortex. Moreover, SBF-SEM analysis of an E12.5 mouse neocortex revealed that, in principle, the same key finding, that is, ETC processes entering neural cells via deep plasma membrane invaginations, is observed at this earlier developmental stage.

Our findings have several major implications. First, they suggest that in addition to the well-established bi-directional signaling between the vasculature and the neural cells, which mostly involves secreted factors, there could be another mode of interaction between endothelial cells and neural cells that is based on the contact between the plasma membrane of the endothelial cell process and the invaginated plasma membrane, or perhaps even an endosomal membrane, of the neural cell. The key difference to signaling via secreted factors is that the interaction via membrane contact is characterized by spatial precision, as exemplified by the connexin paradigm (Elias et al., 2010; Penna et al., 2021; Genet et al., 2023).

The second implication we would like to discuss is actually related to the preceding point of discussion and concerns the vesicle clusters that were conspicuously often observed in the vicinity, notably the tip, of the endothelial cell processes that had entered a neural cell (Figures 1G, I, Figure 2B, Figures 3A–E, Figure 6C, and corresponding Supplementary Videos). This observation suggests that the contact between the plasma membrane of the endothelial cell process and the invaginated plasma membrane of the neural cell somehow leads to such vesicle clustering in the nearby neural cell cytoplasm. This, in turn, suggests that some form of intracellular signaling in the neural cell is elicited by the membrane contact between the endothelial cell process and the neural cell. The lower abundance of vesicle clusters associated with intracellular ETC processes at E12.5 compared to E14.5 and the higher proportion of ETC processes ending in shallow grooves rather than deep progenitor plasma membrane invaginations may point to the maturation of these potential signaling centers with development.

It is worth noting that the endothelial cell processes only very rarely contained vesicular structures, whereas the projections formed by neural cells extending in the direction of the ETC body revealed internal membrane profiles (vesicles or endoplasmic reticulum). Interestingly, in the progenitor cytoplasm, the vesicles clustering around tip cell processes were not delimited by a surrounding membrane, as is the case for multivesicular bodies, but were a loose aggregation of vesicles of slightly heterogeneous size. It has previously been reported (Errede et al., 2014) that the CXCL12-positive vesicle cluster accumulates at contact points between neural progenitor cells and blood vessels and co-localizes with connexin 43. The direct contact of the vessel wall may have a similar vesicle-inducing effect as the tip cell processes. It is intriguing that the vesicles surrounding the ETC process tip often cluster at the cell extremity (like the apical chimeric protrusions in the ventricle shown in Figure 3) in an advantageous position for the formation of extracellular particles. A large variety of extracellular vesicles have been identified in recent years, which can convey important signaling functions (Holm et al., 2018; Gustafson and Gammill, 2022; Jeppesen et al., 2023). It is conceivable that the vesicle clusters described here form a novel variant of one of these extracellular particle classes—a question that requires further investigation.

Various studies have documented bi-directional signaling between vascular cells and progenitors. However, temporal and spatial differences in these interactions have been reported (Vasudevan et al., 2008; Lange et al., 2016; Paredes et al., 2018; Komabayashi-Suzuki et al., 2019). Accordingly, in the SVZ at mid-neurogenesis, BP expansion is stimulated by the proximity to blood vessels (Javaherian and Kriegstein, 2009; Wagenfuhr et al., 2015). In contrast, in the avascular VZ, APs are maintained in their proliferative state by the interaction with ETCs (Komabayashi-Suzuki et al., 2019; Di Marco et al., 2020). The specificity of these different niches is reflected by different modes of metabolism, induced by bi-directional signaling between HIF-1α- and VEGF-expressing APs and endothelial cells sprouting into the avascular niche of the VZ (Namba et al., 2021; Dong et al., 2022; Andrews and Pearson, 2024). One way these effects on metabolism may be induced, irrespective of oxygenation by already formed blood vessels, is that ETCs might regulate the metabolic state of neural cells by releasing factors like the HIF-1α—downstream factor apelin, which modulates endothelial cell metabolism (Helker et al., 2020).

Third, our findings reveal that endothelial cell processes not only target neural progenitor cells, including APs and BPs, but also newborn neurons. This is surprising since newborn neurons are highly migratory, and maintaining an interaction with slender filopodia might be challenging. However, the filopodia themselves are dynamic structures that can change their length and shape (Leijnse et al., 2022; Blake and Gallop, 2023). The migration of neurons and the dynamics of interkinetic nuclear migration of the progenitor cells may, therefore, be accompanied by dynamic changes of the filopodia. A transient interaction may be sufficient for an exchange of signals. Signaling mediated by filopodia, sometimes called cytonemes, has been reported in invertebrate and vertebrate systems (Prols et al., 2016). Long-range morphogen gradients can be established by this mechanism during embryonic development. As the ETC filopodia are not required for vessel sprouting in general, this may render their role in sensing and sending signals to the environment likely (Phng et al., 2013). In addition to neural cells, pericytes were also found to be targeted by endothelial cell processes. In other words, all cell types present in the developing neocortex at the stages analyzed can be targeted by the endothelial cell processes. It is presumably a plausible extrapolation of our findings that at later stages of neocortex development, astrocytes, oligodendrocytes and microglia may also be targeted by endothelial cell processes.

We also searched for microglial cells in the E14.5 dataset to examine their interaction with neighboring cells (Penna et al., 2021). We found two putative microglia cells, of which one phagocytosed a neuron, whereas the other tightly spread on a blood vessel, as reported previously (Rezaie and Male, 1999; Penna et al., 2021). The latter cell made contact with endothelial cell protrusions, which, however, were shorter than the ETC filopodia (Supplementary Presentation S2).

Fourth, the chimeric processes extending into the ventricular lumen, which consisted of an apical plasma membrane protrusion of an AP ensheathing an endothelial cell process, deserve comment. We find it likely that the endothelial cell process is the driving force in the generation of these chimeric processes, as no such AP protrusions without an endothelial process inside were observed. A molecular basis for this driving force could be the actin cytoskeleton, which is known to have a major role in the generation and dynamics of filopodia (Bornschlogl, 2013; Fischer et al., 2019; Blake and Gallop, 2023).

Fifth, and finally, our findings presumably will be relevant for the development of tissues other than the neocortex. Interactions between the growing vasculature and tissue-intrinsic cells are a common phenomenon, not only in normal development but also in disorders such as cancer (Carmeliet, 2003; Carmeliet and Jain, 2011). It is an exciting perspective that the entry, traversal, and penetration of tissue-intrinsic cells by endothelial cell processes that we report here may, perhaps, be a general feature of tissue development and maintenance with an—as yet to be explored—functional impact.

## Data Availability

All relevant data are contained within the article and are available in the figures and videos of this study. Further inquiries can be directed to the first author.

## Supplementary material

SUPPLEMENTARY VIDEO S1 SBF-SEM stack through the apical ventricular zone of the mouse E14.5 neocortex showing the extremity of a blood vessel with branching filopodia of an endothelial tip cell. A single sectioning plane of this stack is shown in Figure 1B. The endothelial tip cell is labeled in pink in some sectioning planes.

SUPPLEMENTARY VIDEO S2 SBF-SEM stack showing an ETC delimiting a blood vessel and extending processes that traverse the neighboring pericyte and enter and traverse the neighboring progenitor cells. A single sectioning plane of this stack from the embryonic mouse E14.5 neocortex is shown in Figure 1D. The ETC is labeled with magenta dots, a pericyte with white dots, and a BP with blue dots on selected sectioning planes. The ETC process traversing the pericyte and forming a chimeric process through the neighboring BP is marked with white arrows in selected sectioning planes. The ETC process without pericyte ensheathment passing into a neighboring progenitor is marked with a turquoise, slim arrow. Three more examples of processes originating from this ETC are marked with colored arrows: the pink-labeled ETC process is forming a pericyte-chimeric process ending in a vesicle cluster without entering a progenitor cell. The purple short arrow- and the yellow open arrow-labeled processes end in grooves of the nuclear envelope of the BP. The process endings are mostly associated with vesicle clusters.

SUPPLEMENTARY VIDEO S3 SBF-SEM planes through an apical progenitor cell in the apical VZ of the embryonic E14.5 neocortex traversed by an ETC process. Single sectioning planes of this stack are shown in Figures 1G–K. The ETC process is marked by magenta arrows in selected sectioning planes. The vesicle clusters are marked by black arrows in selected sectioning planes.

SUPPLEMENTARY VIDEO S4 SBF-SEM stack through the apical ventricular zone of the mouse E14.5 neocortex showing an ETC process extending filopodia and processes towards which apical and basal progenitors extend protrusions. A single sectioning plane of this stack is shown in Figure 1C. In this SBF-SEM stack, the ETC processes are labeled in magenta on selected sectioning planes. The ETC body is located at the top. Progenitor cells extend processes, deviating from their apical–basal orientation (horizontal) in the vertical direction of the ETC processes.

SUPPLEMENTARY VIDEO S5 SBF-SEM stack of an endothelial tip cell process traversing the cytoplasm of basal progenitors in the intermediate zone of the embryonic mouse neocortex. Selected sectioning planes of this E14.5 SBF-SEM stack are shown in Figure 2A. The ETC process is marked in selected sectioning planes with magenta arrows.

SUPPLEMENTARY VIDEO S6 SBF-SEM stack of an endothelial tip cell process entering, traversing the cytoplasm, and even penetrating newborn neurons in the intermediate zone of the mouse E14.5 neocortex. Selected sectioning planes of this SBF-SEM stack are shown in Figure 2B. The ETC process is marked in selected sectioning planes with magenta dots, neurons with green dots (one shade of green corresponding to one cell), and newborn BP with blue dots. Some image planes are blurred by section debris.

SUPPLEMENTARY VIDEO S7 SBF-SEM stack revealing the long “chimeric” processes in the ventricular lumen of the embryonic mouse neocortex. Selected sectioning planes of this SBF-SEM stack are shown in Figures 3A–E. The ETC processes are labeled by orange and blue arrows on a selected sectioning plane according to the labeling in Figure 3. The long ETC processes extend ensheathed by APs from the apical-most VZ into the ventricular lumen. The long chimeric processes terminate with vesicle clusters formed by the APs around the ETC process tips. The bases of the apical primary cilia of the APs traversed by the ETC processes are marked by white arrows. One sectioning plane was not well-acquired and is replaced by a black layer.

SUPPLEMENTARY VIDEO S8 SBF-SEM stack revealing ETC processes traversing an apical progenitor in anaphase in the embryonic mouse E14.5 neocortex. Two sectioning planes through this anaphase cell are shown in Figures 4A, B. The volume is taken from dataset 2. The anaphase cytoplasm is marked in yellow on selected sectioning planes. The apical plasma membrane is outlined in yellow on some sectioning planes. The chromatin is labeled in blue. Centrioles are labeled by turquoise/white or turquoise/magenta dots. The ETC processes are labeled by magenta lines. The contrast values of the image tiles are not matched perfectly (border between tiled images to the right).

SUPPLEMENTARY VIDEO S9 SBF-SEM stack revealing an ETC process traversing the cytoplasm of a newborn basal progenitor in the VZ of the E12.5 mouse neocortex. A single plane of the SBF-SEM stack is shown in Figure 6B. The extremity of a blood vessel is surrounded by an ETC (magenta). One ETC process (magenta arrows) traverses a newborn BP (small blue dots) which is protruding in the direction of the ETC process (blue arrows indicate the BP protrusion).

SUPPLEMENTARY VIDEO S10 SBF-SEM stack revealing ETC processes traversing the cytoplasm of progenitors and forming chimeric processes with vesicle clusters in the VZ of the E12.5 mouse neocortex. A single plane of the SBF-SEM stack is shown in Figure 6C. Close to a blood vessel, an ETC process (magenta) is branching into two branches (in the video volume on top of each other), of which one is traversing the cytoplasm of a newborn BP (marked by blue dots), whereas the other branch is traversing the cytoplasm of an AP (marked by yellow dots), forming a chimeric process. Vesicle clusters are present in the progenitor cytoplasm next to the ETC processes.

SUPPLEMENTARY VIDEO S11 An ETC process traverses the cytoplasm of an AP, forming a chimeric process into the ventricular lumen of the E12.5 mouse neocortex. The single planes of the SBF-SEM stack are shown in Figure 6D. The base of the apical primary cilium of the AP, which is extending a chimeric process with the ETC process (magenta) into the ventricular lumen is marked with a white arrow.

SUPPLEMENTARY VIDEO S12 An ETC process in the intercellular space in the VZ of an E12.5 mouse neocortex is touching the plasma membrane of progenitor cells. Selected planes of the SBF-SEM stack are shown in Figure 6E. An ETC process (magenta lines) is touching APs in plasma membrane grooves (magenta arrowheads). The APs have duplicated centrioles in deep plasma membrane invaginations. (Yellow labels are segmentations of neighboring cells not of relevance for this figure).

SUPPLEMENTARY DATA SHEET S1 Quantification of the number, location and destination of ETC processes in two E14.5 and one E12.5 SBF-SEM datasets. **Sheet 1:** Mouse E14.5 data. Sheet 1: Location of ETC process endings. For the individual endothelial tip cells (listed by their cell ID in the Fiji/TrakEM segmentation file) the number of all tracked processes (total processes) and the location of their endings according to their position in the intercellular space (intercell #) or having entered a cell (intra all #) are given. For processes which have entered a cell, it is listed whether they are extending into the ventricular lumen (intra ventricle #), or forming a chimeric process between neural/mural cell and ETC (intra chimeric #). The location of the ETC cell body in the VZ (black cell ID) or SVZ/IZ (red cell ID) is distinguished. Some ETCs were not completely contained in the SBF-SEM stacks or the processes were not completely tracked. Sheet 1: Percentages. The number of ETC process endings at the respective location (intercellular or intracellular, or both (all)) are summarized, and percentages of endings in a given location, over all endings in the respective layer (VZ or SVZ/IZ), listed. For the ETC endings in the VZ, the percentages are listed separately for the two datasets (VZ1 or VZ2) in the right half of the table. Sheet 1: Type of cells with ETC process contact. For the intracellular ETC process endings from each ETC (by cell ID, red: in SVZ/IZ), the type of their final target cell is listed: apical progenitor (AP), newborn basal progenitor (aBP), basal progenitor (BP), neuron (N), pericyte (peri) or putative microglia cell (MG). In the third column (chimeric), these cells are listed which are penetrated or traversed by the process between the ETC cell body and the ending on the target cell. **Sheet 2:** mouse E12.5 data. Sheet 2: Location of ETC process endings. For the individual endothelial tip cells (listed by their cell ID in the Fiji/TrakEM segmentation file), all tracked processes are listed according to the location of their endings in the intercellular space (intercellular), on the plasma membrane of a progenitor (touching) or intracellular (intra). For the processes which ended either on a progenitor plasma membrane or having entered a target cell, the type of the target cell is listed: AP, mitotic AP (anaphase/prometaphase, or with late telophase (young cilium (C)), newborn BP (aBP), or pericyte (peri). For some progenitors, the location of the primary cilium on the apical or basolateral plasma membrane (aC or blC) and the number of centrioles is given (duplicated centrioles: 4C, non-duplicated: 2C) for classification. Sheet 2: Quantification of ETC cell end location. For the two ETCs in the mE12.5 VZ contained in the dataset with multiple tracked processes, the number of processes (total #) is given and subdivided according to the location of the ending by number of cells and percentage over all processes of this individual cell (by cell ID) or over both cells (all). Sheet 2: Cell types targeted by ETCs. The number of intracellular or touching ETC process endings from the two ETCs in the respective location is given. The mitotic APs are already contained in the number of intracellular APs (therefore the number is gray).

SUPPLEMENTARY PRESENTATION S1 Models from the SBF-SEM segmentations corresponding to the panels in Figures 1–5 and Figure 6, and TEM images corresponding to Figure 3.

SUPPLEMENTARY PRESENTATION S1-1C Model view from the SBF-SEM segmentation through an ETC extending filopodia and processes in the apical ventricular zone of the mouse E14.5 neocortex with apical progenitors and a newborn basal progenitor extending protrusions towards the ETC processes. A single sectioning plane of the corresponding SBF-SEM stack is shown in Figure 1C and a SBF-SEM stack in Supplementary Video S4. The outlines of apical progenitors are shown in yellow, of the newborn basal progenitor in blue. Only the ventricular third of the cells is shown.

SUPPLEMENTARY PRESENTATION S1-1F Model view from the SBF-SEM segmentation through an ETC delimiting a blood vessel and extending processes which traverse the neighboring pericyte and enter the neighboring newborn basal progenitor cell. Two tilt views of this model are shown in Figure 1F. The position is corresponding to Figure 1D and Supplementary Video S2.

SUPPLEMENTARY PRESENTATION S1-1L (low) Model view from the SBF-SEM segmentation through an apical progenitor cell in the apical VZ of the mouse E14.5 neocortex traversed by an ETC process. Single sectioning planes through these cells are shown in Figures 1G–K and a SBF-SEM stack in Supplementary Video S3. The outline of the AP is shown in yellow, stretching from the ventricle (left) to the basal lamina (right). From the duplicated centrioles, and the position of the nucleus in the apical VZ, the AP can be assumed to be in the G2-phase of the cell cycle. The process of the magenta endothelial traverses the lateral side of this AP as seen at higher magnification in Supplementary Presentation S1-1L (high).

SUPPLEMENTARY PRESENTATION S1-1L (high) Model view from the SBF-SEM segmentation through an apical progenitor cell in the apical VZ of the mouse E14.5 neocortex traversed by an ETC process. Single sectioning planes through these cells are shown in Figures 1G–K and a overview in Supplementary Presentation S1-1L (low). The process of the magenta endothelial traverses the lateral side of the apical progenitor in G2-phase (in yellow). The endothelial cell is not modelled completely.

SUPPLEMENTARY PRESENTATION S1-2C Model view from the SBF-SEM segmentation through the endothelial tip cell traversing a basal progenitor in the intermediate zone of the mouse E14.5 neocortex. A selected plane of this model is shown in Figure 2C and a SBF-SEM stack in Supplementary Video S5. The BP is shown in blue, the ETC in magenta. The magenta arrow points to the position where the ETC process is traversing the BP.

SUPPLEMENTARY PRESENTATION S1-2D Model view from the SBF-SEM segmentation through the endothelial tip cell entering newborn neurons and ending in a vesicle cluster surrounded by a newborn basal progenitor in the intermediate zone of the mouse E14.5 neocortex. A selected plane of this model is shown in Figure 2D and a SBF-SEM stack in Supplementary Video S6. The newborn neurons are labelled in light and dark green, the newborn BP in blue. The ETC cell is modelled in magenta. The ventricle is to the left, basal lamina to the right.

SUPPLEMENTARY PRESENTATION S1-3F Model view from the SBF-SEM segmentation of the embryonic mouse E14.5 neocortex visualizing the blood vessel network with ETCs and apical progenitors forming “chimeric” processes with the ETCs in the ventricular lumen. A selected plane of the region shown in this model is shown in Figure 3F and Supplementary Video S7. The ETCs are labelled in magenta, the blood vessel lumen in purple. The AP with ETC-AP–chimeric processes into the ventricular lumen are displayed in yellow. Apical/ventricle is left, the basal lamina to the right.

SUPPLEMENTARY PRESENTATION S1-I3 Long “chimeric” processes in the ventricular lumen of the embryonic mouse E14.5 neocortex contain the cytoplasm and plasma membrane of the endothelial tip cell surrounded by cytoplasm of the progenitor cell limited by an inner and outer plasma membrane. Transmission electron micrographs through ultrathin sections of an mE14.5 neocortex illustrating the triple membrane profiles in “chimeric” processes in the ventricular lumen. The sample preparation was performed as described for the SBF-SEM datasets. The micrographs were acquired on a Thermo Fisher/FEI Morgagni transmission electron microscope at 80 KV and with a Emsis Morada camera. **(A)** Overview with the ventricular surface of the cortex (right) and two sectioning planes through an “chimeric” process in the lumen. The host cell being an apical progenitor is an assumption. Magenta dots indicate the ETC cytoplasm, yellow dots the host cell cytoplasm. **(B)** Higher magnification of the cross-sectional view of the ETC process in A, displaying the ETC plasma membrane (solid magenta arrow), adjacent to the progenitor “inner” plasma membrane (yellow open arrow) of the progenitor protrusion, which together with the “outer” plasma membrane (yellow solid arrow) encloses the progenitor cytoplasmic sheath. **(C)** Subsequent section of the “chimeric” process shown in A and B (located in between the two fragments through the “chimeric” process in A), displaying the triple membrane structure and a rare vesicle inside the ETC cytoplasm. V: ventricle. Scale bar in A: 1 µm, in B and C: 0.1 µm.

SUPPLEMENTARY PRESENTATION S1-4C Model view from the SBF-SEM segmentation through the ETC processes traversing an apical progenitor in anaphase in the embryonic mouse E14.5 neocortex. Two selected planes of the volume contained in this model are shown in Figures 4A, B and Supplementary Video S8. The anaphase cell (in yellow) with chromatin (blue) and traversing ETC processes (magenta) is shown at lower magnification in Supplementary Presentation S1-4D. The apical plasma membrane of the mitotic AP contacting the ventricular lumen is shown in bright yellow. Centrioles are shown in white.

SUPPLEMENTARY PRESENTATION S1-4D Model view from the SBF-SEM segmentation through the ETC processes traversing an apical progenitor in anaphase in the embryonic mouse E14.5 neocortex. Two selected planes of the volume contained in this model are shown in Figures 4A, B and Supplementary Video S8. Lower magnification of the volume shown in Supplementary Presentation S1-4C, showing an ETC (magenta) which sends processes into the apical anaphase AP (yellow) at the ventricular surface. The apical plasma membrane of the mitotic AP is shown in bright yellow, centrioles in white.

SUPPLEMENTARY PRESENTATION S1-6A Model view from the SBF-SEM segmentation of the embryonic mouse E12.5 neocortex visualizing ETCs with long slender filopodia entering progenitors. Selected planes of the volume shown in this model are shown in Figure 6 and Supplementary Videos S9–S12. An ETC with many filopodia is labelled in magenta in the center of the volume, another ETC is only partially modelled (magenta separate line). The blood vessel lumen in purple. An AP (yellow) and two newborn BPs (blue) are displayed which are traversed by ETC processes. Apical/ventricle is left, the basal lamina to the right.

SUPPLEMENTARY PRESENTATION S2 Interaction of microglia cells.

SUPPLEMENTARY PRESENTATION S2-Model Model view from the SBF-SEM segmentation of the embryonic mouse E14.5 neocortex visualizing the blood vessel network with ETCs and microglia cells interacting with neural cells. Model view of the ETCs and blood vessels (magenta) contained in the SBF-SEM volume through the mouse E14.5 neocortex showing two microglia cells (white). The microglia cell to the right is contacting APs (yellow colors), of which one (darker yellow) is in prometaphase and engulfing a neuron (green). The microglia cell to the left is tightly associated with a blood vessel. From each microglia cell a volume is shown in Supplementary Videos S1, S2.

SUPPLEMENTARY PRESENTATION S2-Video 1 SBF-SEM stack revealing microglia protrusions tightly associated with endothelial cells in the VZ of the embryonic mouse E14.5 neocortex. SBF-SEM stack of endothelial cells (magenta dots on one endothelial cell) surrounding a blood vessel (to the left) in the VZ. The endothelial cells are extending short protrusions into an adjacent microglia cell (left cell in Supplementary Presentation S2-Model) (white dots and color). Vesicles clusters are present where progenitors contact the microglia or endothelial cell.

SUPPLEMENTARY PRESENTATION S2-Video 2 SBF-SEM stack revealing microglia protrusions engulfing a neuron and contacting APs in the intermediate zone of the embryonic mouse E14.5 neocortex. SBF-SEM stack with wide-spread protrusions of a microglia cell (white dots and color) engulfing a neuron (green lines and color). The microglia cell is contacting and partly surrounding basal processes of an interphase AP (lighter yellow) and of an AP in prometaphase (darker yellow).
