# Supplementary material for: High-resolution 3D ultrastructural analysis of developing mouse neocortex reveals long slender processes of endothelial cells that enter neural cells
Source: Front Cell Dev Biol. 2024 Mar 4;12:1344734. doi: 10.3389/fcell.2024.1344734 (PMC10945550; doi:10.3389/fcell.2024.1344734)
Supplement: Supplementary file 5 [file Presentation1.PPTX]

## Slide 1
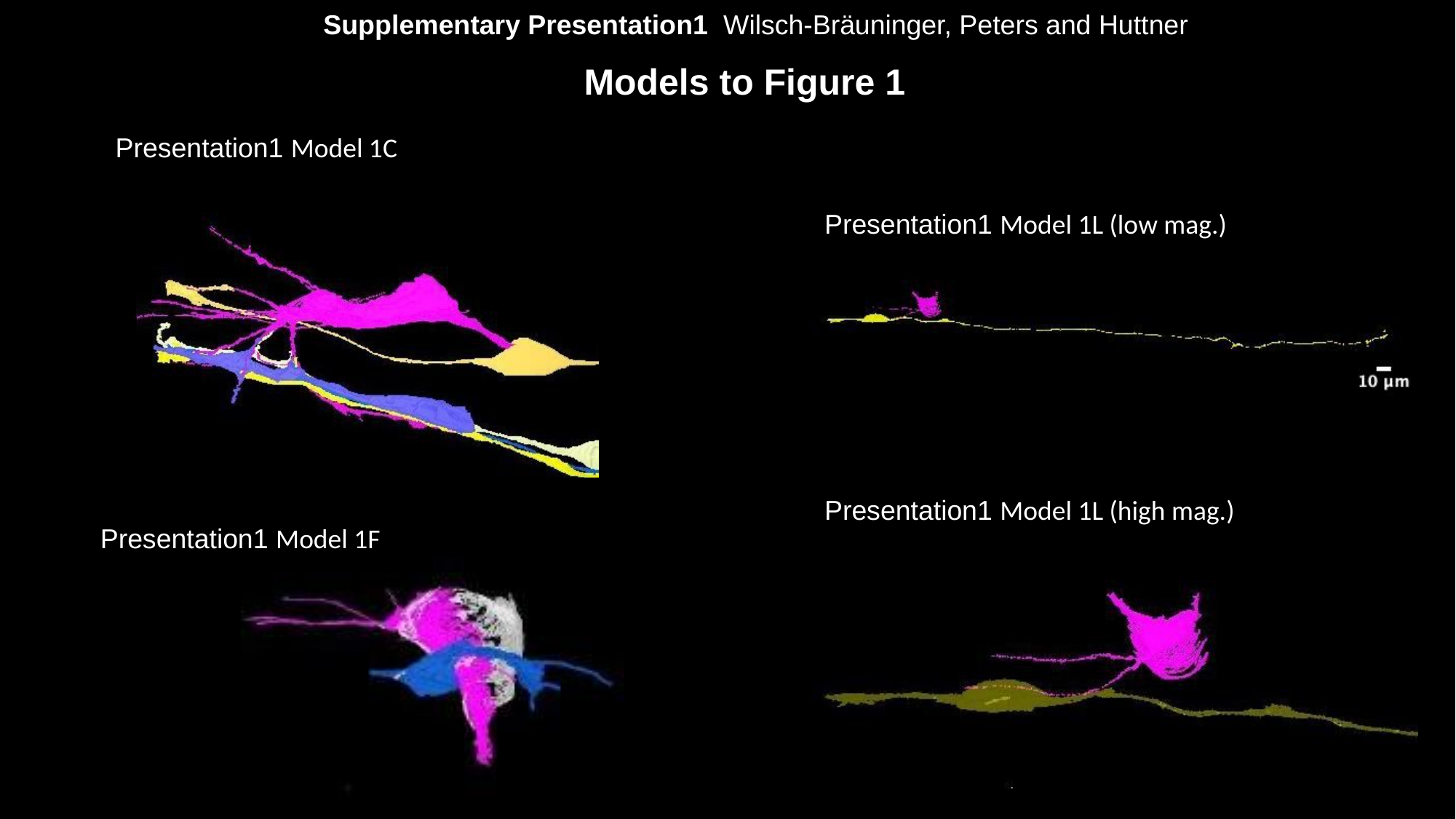

Supplementary Presentation1 Wilsch-Bräuninger, Peters and Huttner
Models to Figure 1
Presentation1 Model 1C
Presentation1 Model 1L (low mag.)
Presentation1 Model 1L (high mag.)
Presentation1 Model 1F

## Slide 2
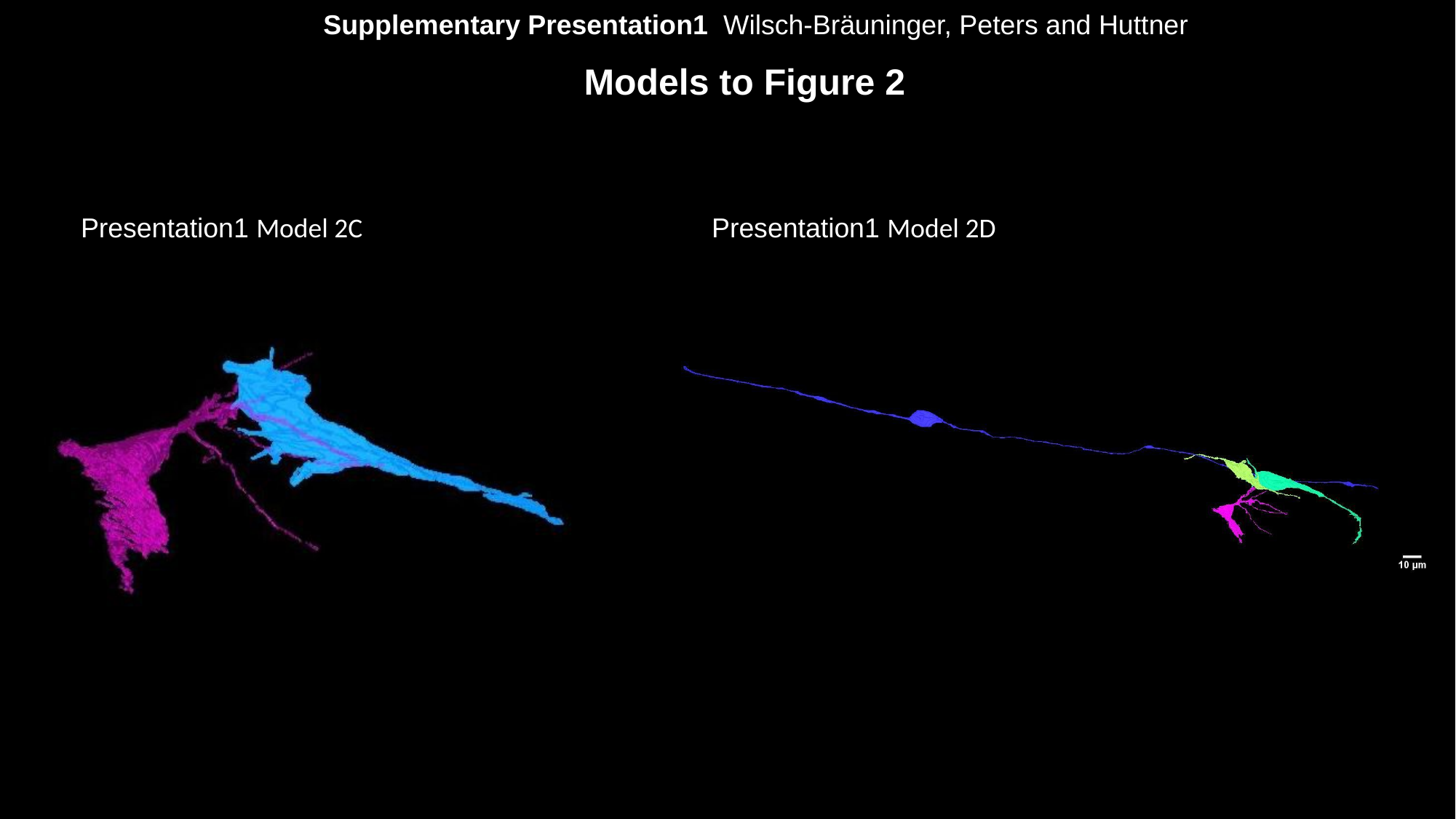

Supplementary Presentation1 Wilsch-Bräuninger, Peters and Huttner
Models to Figure 2
Presentation1 Model 2C
Presentation1 Model 2D

## Slide 3
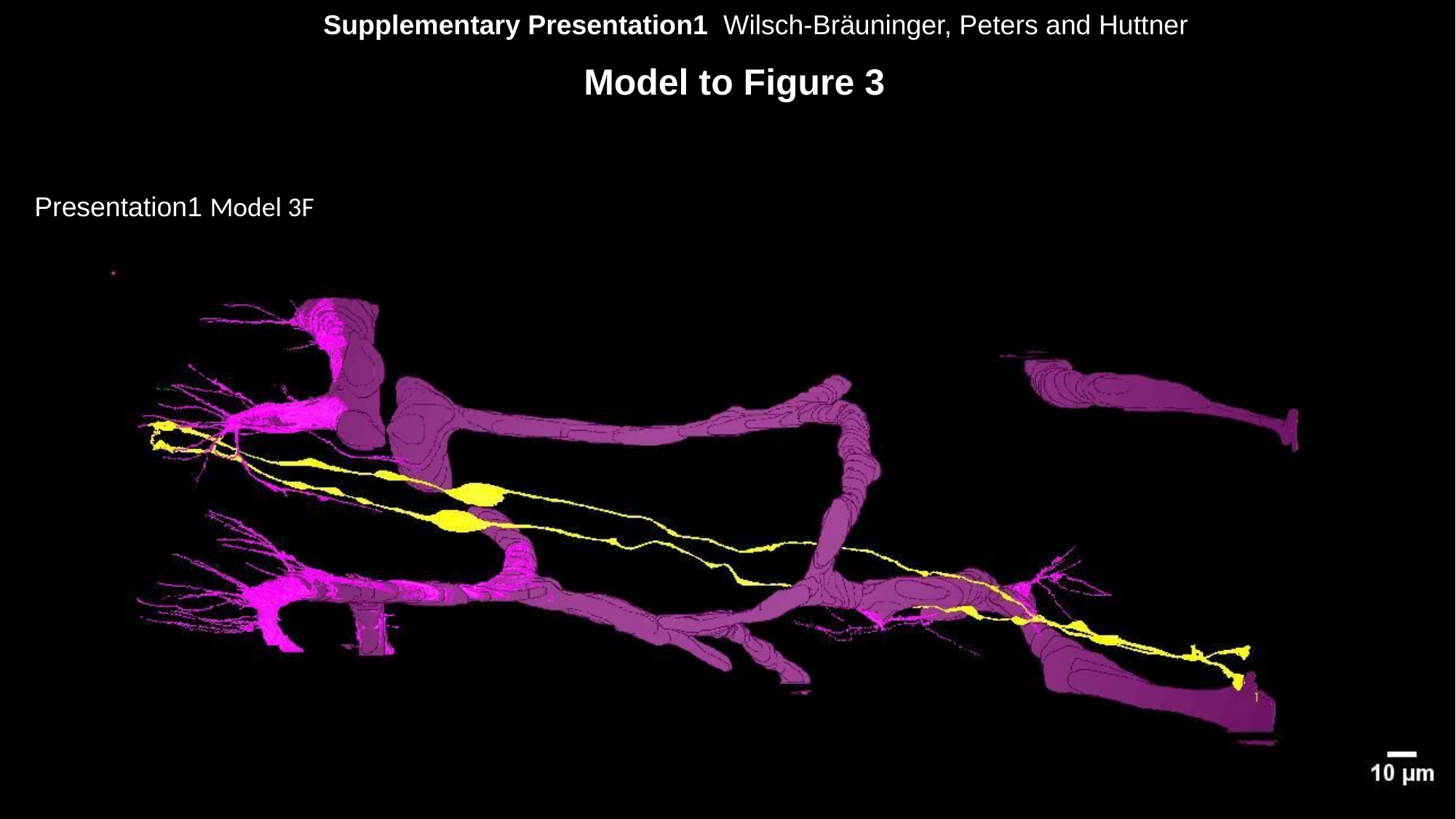

Supplementary Presentation1 Wilsch-Bräuninger, Peters and Huttner
Model to Figure 3
Presentation1 Model 3F

## Slide 4
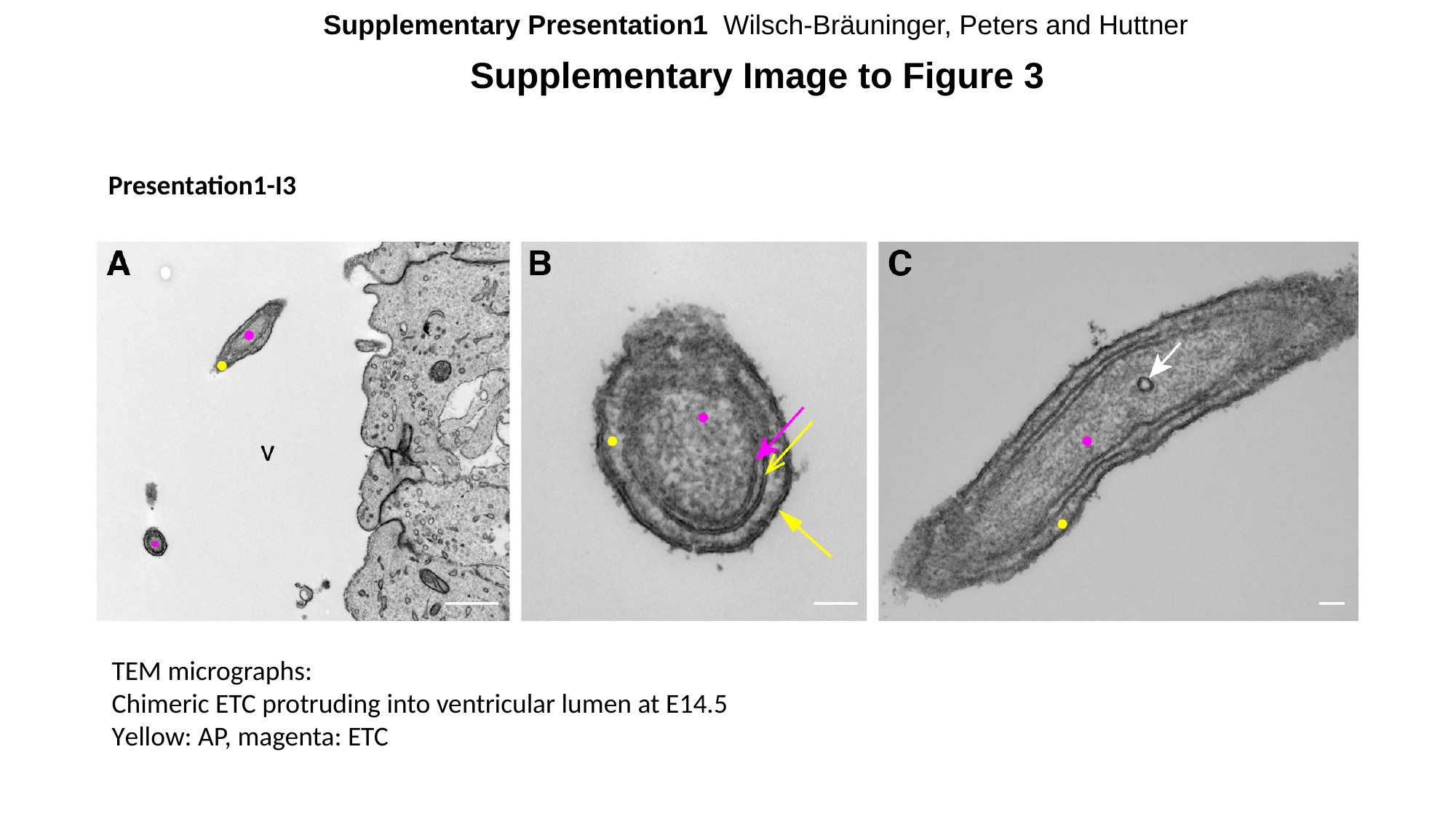

Supplementary Presentation1 Wilsch-Bräuninger, Peters and Huttner
Supplementary Image to Figure 3
Presentation1-I3
TEM micrographs:
Chimeric ETC protruding into ventricular lumen at E14.5
Yellow: AP, magenta: ETC

## Slide 5
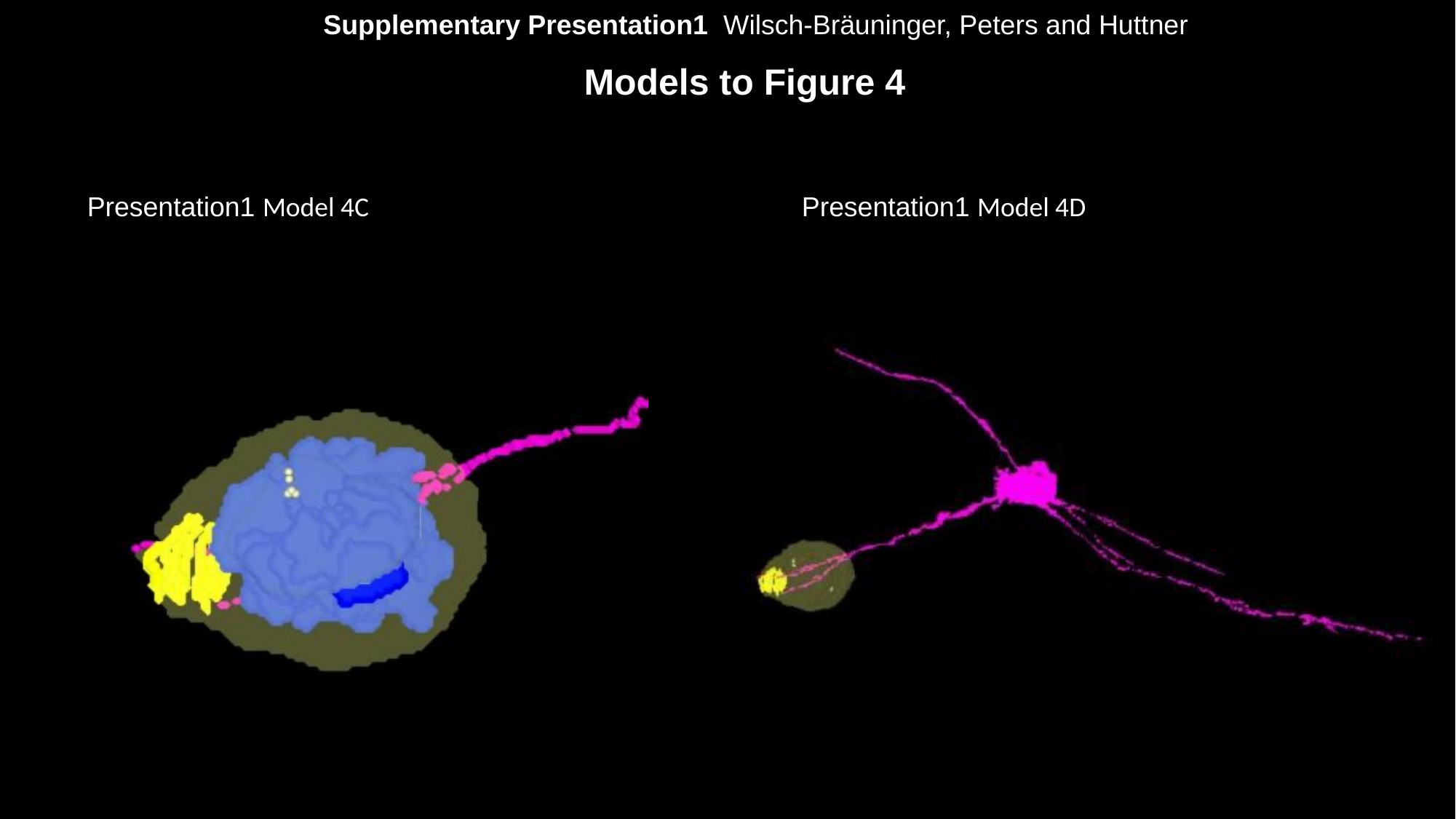

Supplementary Presentation1 Wilsch-Bräuninger, Peters and Huttner
Models to Figure 4
Presentation1 Model 4C
Presentation1 Model 4D

## Slide 6
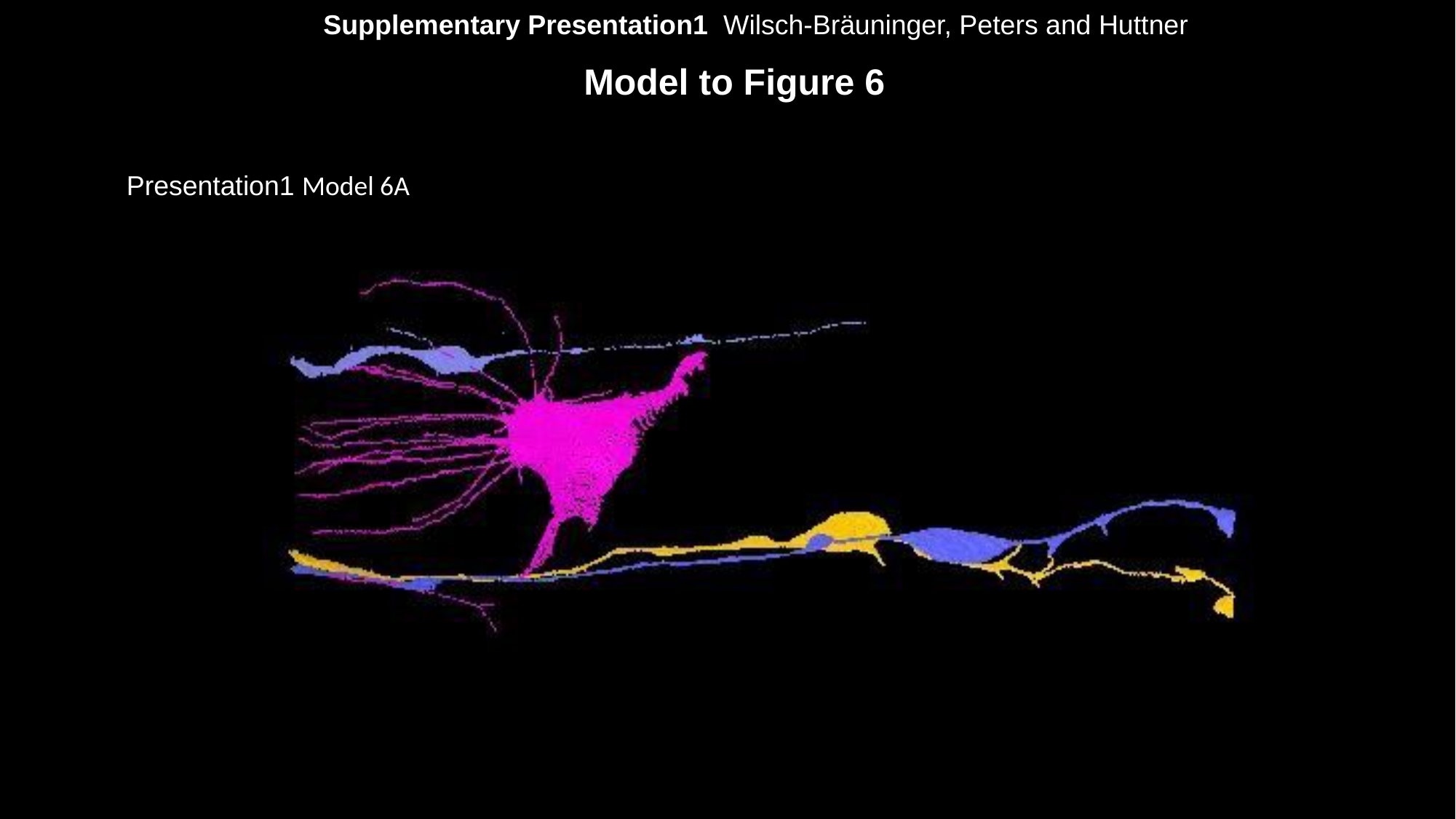

Supplementary Presentation1 Wilsch-Bräuninger, Peters and Huttner
Model to Figure 6
Presentation1 Model 6A
