# Supplementary material for: High-resolution 3D ultrastructural analysis of developing mouse neocortex reveals long slender processes of endothelial cells that enter neural cells
Source: Front Cell Dev Biol. 2024 Mar 4;12:1344734. doi: 10.3389/fcell.2024.1344734 (PMC10945550; doi:10.3389/fcell.2024.1344734)
Supplement: Supplementary file 9 [file Presentation2.PPTX]

## Slide 1
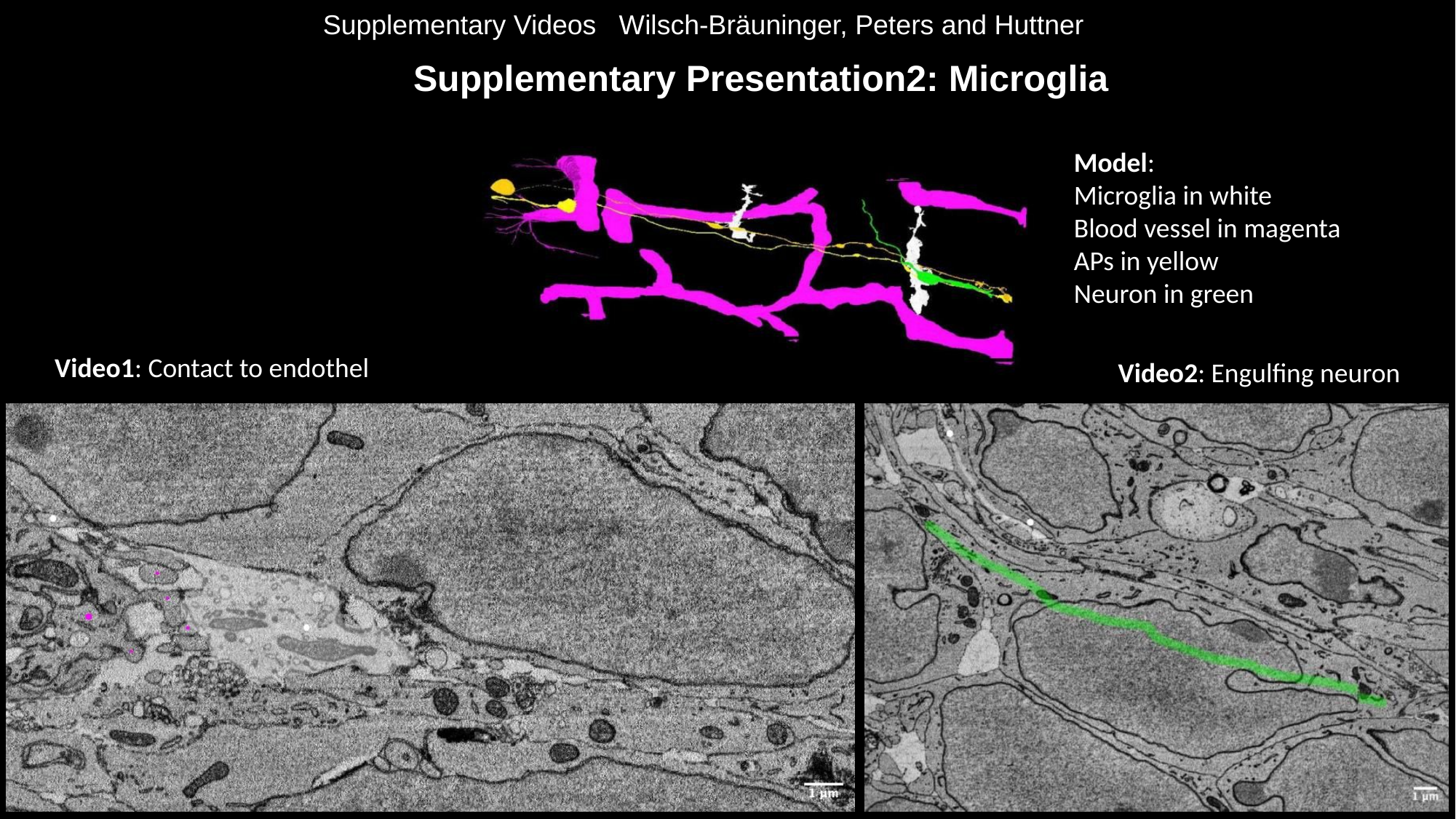

Supplementary Videos Wilsch-Bräuninger, Peters and Huttner
Supplementary Presentation2: Microglia
Model:
Microglia in white
Blood vessel in magenta
APs in yellow
Neuron in green
Video1: Contact to endothel
Video2: Engulfing neuron
